# Flexural Tensile Strength of Concrete with Synthetic Fibers

**DOI:** 10.3390/ma14164428

**Published:** 2021-08-07

**Authors:** Julia Blazy, Łukasz Drobiec, Paweł Wolka

**Affiliations:** 1 Faculty of Civil Engineering, Silesian University of Technology, Akademicka 5, 44-100 Gliwice, Poland; lukasz.drobiec@polsl.pl; 2 Astra Technologia Betonu Sp. Z.O.O., 83-010 Straszyn, Poland; pawel@astra-polska.com

**Keywords:** fiber-reinforced concrete, synthetic fibers, three-point bending test, crack mouth opening displacement, crack tip opening displacement, deflection, flexural tensile strength, toughness, uniaxial tensile test, Digital Image Correlation

## Abstract

Fiber reinforcement is currently most often used in floors, railway sleepers, prefabricated structural elements such as slabs, beams and tanks, and in small architecture elements. Designing elements or structures made of fiber-reinforced concrete requires knowledge of its basic mechanical parameters. In the case of concretes with metallic fibers, the literature can find many tests and standard guidelines regarding compressive, flexural, tensile strength and fracture energy. The properties of concretes with non-metallic fibers are slightly less recognized, especially concretes with new types of polymer fibers. Additionally, the lack of standardized methods of testing concrete with polymer fibers make their application much more difficult. In the article, the possibility of using the EN 14651 standard to assess the flexural tensile strength of concrete with the addition of 2.0 and 3.0 kg/m^3^ of synthetic fibers with different geometry and form was presented. There was a 5.5–13.5% increase in the flexural tensile strength depending on the mixture type. Moreover, in the case of fiber-reinforced concretes, the ductility was enhanced and the samples were characterized by significant residual flexural tensile strengths. Additionally, from the workability tests it was concluded that after the incorporation of fibers, the consistency class decreased by one, two or three. Nevertheless, the compressive strengths of concrete with and without fibers were very similar to each other, and varied from 58.05 to 61.31 MPa. Moreover, it was concluded that results obtained from three-point bending tests significantly differed from empirical formulas for the calculation of the flexural tensile strength of fiber-reinforced concretes with dispersed steel fibers present in the literature. As a result, the new formula determined by the authors was proposed for concrete with polymer fibers with a nominal fiber content ≤1.0% and slenderness of up to 200. It must be mentioned that the formula gave a very good agreement with studies presented in different literature positions. In addition, an attempt was made to evaluate the strengths of tested mixes in accordance with the Model Code 2010. However, it occurred that the proposed fiber-reinforced concrete mixtures would not be able to replace traditional reinforcement in a form of steel bars. Furthermore, in uniaxial tensile tests, it was not possible to determine the σ–w graphs, and received results for maximum tensile strength did not show the clear influence of fibers incorporation on concrete. Then, the fracture energy enhancement (from about 16 to 22 times) and dependencies: crack mouth opening displacement–deflection; crack mouth opening displacement–crack tip opening displacement; and crack tip opening displacement–deflection were analyzed. Finally, the results from flexural tensile tests were compared with measurements of the surface displacement field obtained through the Digital Image Correlation technique. It was concluded that this technique can be successfully used to determine the crack mouth and crack tip opening displacements with very high accuracy.

## 1. Introduction

Fiber-reinforced concrete (FRC) is obtained by adding steel or non-metallic, e.g., synthetic fibers to the concrete mix. Metallic fibers are often used to significantly improve the mechanical properties of concrete, and when protection against cracks is very crucial. Therefore, steel fibers are used in the production of, among others, industrial floors [1,2], prefabricated constructional elements [3], prestressed elements [4,5] and linings of tunnels [6]. Moreover, the recent investigations on the application of FRC highlight the possibility of using them to manufacture railway sleepers [7,8]. Synthetic fibers also improve the properties of concrete, but to a lesser extent than steel fibers [9,10]. They are especially used because of their increased residual tensile strength, and thus better ductility compared to concrete without fibers [11]. Additionally, due to the fact that the fibers bridge cracks and their amount in concrete can be much higher than for steel fibers because their weight is smaller, they successively limit the width of cracks resulting from concrete shrinkage [12]. FRC with synthetic fibers is most often used in industrial floors, traffic surfaces, concrete slabs, tanks for liquids, elements of maritime infrastructure, culverts, as shotcrete and in elements of small architecture [13,14]. It is also noteworthy that synthetic fibers can be used in places exposed to water, as opposed to steel fibers, as it would not cause their corrosion [14]. The idea of durability enhancement due to the application of non-metallic reinforcement, which additionally limits the crack formations, is discussed in many studies, e.g., [15,16,17,18]. Sometimes both steel and synthetic fibers are added to the concrete, in order to obtain a mix with optimized properties and better performance [19].

The mechanical characteristics of FRC depend on the properties of the concrete matrix, but also on the material, dimensions (l_f_-fiber length; d_f_-fiber diameter), type and nominal volume content (V_f_) of fibers in the concrete. In addition, the bonding mechanism between the fiber and concrete also has an impact, i.e., whether the fibers are monofilament or fibrillated. Synthetic fibers can be divided into micro-and macrofibers. They are distinguished by their diameter: the microfibers have d_f_ < 30 mm and the macrofibers have d_f_ > 30 mm [20]. Their function is also different: microfibers prevent from microcracks occurring at the beginning of the concrete hardening process and increase tensile strength, and macrofibers often play an additional load-bearing function, protect against macrocracks and increase concrete ductility [4]. Furthermore, concrete is often formed with the addition of these two types of fibers to obtain a mixture that both replaces the traditional steel bar reinforcement and counteracts shrinkage [21].

The incorporation of synthetic fibers into the concrete mix influences its properties in various ways. Typically, the modulus of elasticity and compressive strength are similar to those for concrete without fibers. On the other hand, the fibers will have a very positive effect on ductility, toughness and freeze-thaw resistance, and will significantly reduce shrinkage, crack width and spalling during fire [14,22]. Properties such as abrasion resistance, tensile strength and flexural tensile strength will also be improved [14]. However, it should be noted that the influence of the addition of fiber in concrete largely depends on the workability of the concrete mix [23]. Namely, if it significantly deteriorates, the mechanical properties of concrete may be degraded, especially when its porosity, permeability and/or the amount of water absorbed will increase.

In order to test concrete in tension, the literature usually proposes two test methods: uniaxial tensile test (UTT) and three-point bending test (3PBT). However, UTT must be performed under very well-controlled conditions, using specialized equipment, without eccentricities, and on an ideal sample [24,25]. Otherwise, the test may be unstable and the results may be incorrect [25]. Moreover, UTT is very time-consuming, and depends on the interaction of the machine with the sample [24]. The easier and more popular test is 3PBT. Additionally, Model Code 2010 [26] and Technical Report 34 [27] state that in order to design FRC elements, it is necessary to determine the residual flexural tensile strength using 3PBT in accordance with EN 14651 [28].

Moreover, the Digital Image Correlation (DIC) technique is increasingly used to investigate the behavior of FRC. Babski et al. [29] analyzed the deformations of the ultra-high-performance steel FRC samples and the crack propagation under tensile loading using the DIC system. In [30], fracture process zone development during the wedge-splitting test of concrete reinforced with recycled and industrial steel fibers applying the DIC system was examined. There are studies in which DIC was used to measure fracture parameters during 3PBT of plain concrete [31,32]. Experiments done by Manning et al. [33] focused on applying the DIC technique to study stress-strain behavior of prestressed channel girders reinforced with steel fibers. Additionally, in [34], slender steel FRC beam members under shear loading were monitored by the DIC system. As can be seen, many of the available studies focus on analyzing the concrete using the DIC system for plain concretes [35,36], traditionally reinforced concretes by steel bars [35,37] or steel FRC, less on synthetic FRC. Namely, in [38], a study on residual compression behavior of polypropylene FRC subjected to moderate temperature was performed. On the other hand, Bertelsen et al. [39] focused on plastic shrinkage while testing samples with micro synthetic fibers. Finally, under 3PBT, the polyolefin FRC were investigated in [40] by Rucka et al., and in [41] by Bhosale and Prakash. Furthermore, one of the reasons for such big popularity of this optical technique is its non-destructive and non-contact character. Secondly, the technique allows recoding the damage evolution continuously and in real time. Finally, thanks to the DIC system, a large number of samples can be studied as the visualized surface deformation measurements are obtained quickly by successive post-processing of digital images.

Regarding the research significance, it must be noted that even though the EN 14651 standard [28] applies to concrete reinforced with metallic fiber, in the article it was proofed that the standard can be successively used when concrete is reinforced by synthetic fibers. In the article, three types of non-metallic fibers were added to concrete mix in amounts of 2.0 and 3.0 kg/m^3^, for which the limit of proportionality and residual strengths were described. Furthermore, the proposition of new formula, despite the one presented in the EN 14651 standard [28] for metallic fibers, to describe the relationship between deflection and crack mouth opening displacement for synthetic fibers was introduced. Other dependencies such as crack mouth opening displacement-crack tip opening displacement and crack tip opening displacement-deflection were analyzed to help other researchers in the case when only one parameter would be measured during the 3PBT, and the calculation of residual strengths would be required. Additionally, formulas presented in the literature for the calculation of flexural tensile strength for FRC are usually dedicated to reinforcement with metallic fibers [42,43,44,45]. This study will fill the knowledge gap when it comes to the estimation of bending strength for polymer FRC with a nominal fiber content ≤ 1.0% and slenderness of up to 200. Furthermore, an attempt was made to evaluate the tensile strengths in uniaxial tensile tests on samples significantly bigger than the one usually used in this kind of test [45,46]. It must be mentioned that the proprietary set-up was designed and used to execute this type of test. Concerning the classification of FRC according to Model Code 2010 [26], only a limited number of articles was found. Namely, in [46] Carlesso et al. classified the post-cracking strengths of FRC, but with a higher amount of polypropylene fibers from 5.0 to 10.0 kg/m^3^. On the other hand, for similar V_f_ the classification is done in [47]. Other research [48] also mentioned the toughness class, but for steel FRC. Moreover, since little work has been done using the DIC technique for polymer FRC subjected to 3PBT, the current study will help to enhance the knowledge in this area. The conclusions from the comparison of results from clip gauges and the DIC system will be beneficial for other researchers. It must be also mentioned that Glinicki in [13] performed similar studies with 2.0–3.9 kg/m^3^ of macro synthetic fibers for industrial floors. However, they covered just the compressive strength test and four-point bending test according to ASTM C 1018-94 standard [49], so were different than presented in the current research. An extensive study on the effect of polypropylene fibers on the properties of concrete is also presented in [50]. Nevertheless, it did not deal with the issue of residual flexural tensile strengths and fracture energy. In [46], 3PBT was performed according to [29], though the amount of fibers was higher than the one proposed in the current study and was equal to 5.0–10.0 kg/m^3^. Moreover, the work focused more on the fatigue behavior of polymer FRC. To sum up, according to the author’s knowledge and prepared Table 1, there is no research that simultaneously covers aspects of influence of synthetic fibers on workability; compressive, flexural, tensile strength and toughness, together with classification of post-cracking strengths according to Model Code 2010 [26] and complex study on empirical equations to explain the improvement in bending strength for no-metallic fibers.

## 2. Materials and Methods

### 2.1. Materials

The tests were carried out with the use of three types of synthetic fibers, marked: PM, PD and FF. The scope of the research included the preparation of six concrete mixes: reference-without fibers (PC) and with the addition of: 3.0 kg/m^3^ of PM fibers (PM_3); 2.0 kg/m^3^ of PM fibers (PM_2); 3.0 kg/m^3^ of PD fibers (PD_3); 2.0 kg/m^3^ of PD fibers (PD_2); and 2.0 kg/m^3^ of FF fibers (FF_2). The fibers added to the concrete mix are characterized in Table 2. All fibers had similar tensile strength (f_t_), chemical resistance, and their density was equal to 0.91 g/cm^3^, melting point 160–170 °C. On the other hand, they differed in dimensions and form, e.g., PM and FF fibers were longer and slender than PD fibers and were added to the mixture in the form of bundles, which disintegrate during mixing. In addition, Portland cement type I of the strength class 42.5 with high early strength (R)-CEM I 42.5R meeting the requirements of EN 197-1 [57] was used. Moreover, coarse pebble aggregate with a diameter of 2–8 mm was used, and as a fine-grained aggregate sand with a maximum diameter of 2 mm. In order to ensure proper workability, a superplasticizer based on a modified acrylic polymer–MAPEI Dynamon SX 08 was added to the mixture. Pure water from the water supply network was used. The water-cement ratio (w/c) was equal to 0.50. Each batch of mixes was prepared in a single excipient. All mixes were made on the same day, at a similar temperature and humidity. The composition of all concrete mixes is shown in Table 3. It is also worth noting that it was constant, and the only variable was nominal volume content V_f_.

**Figure 1:**
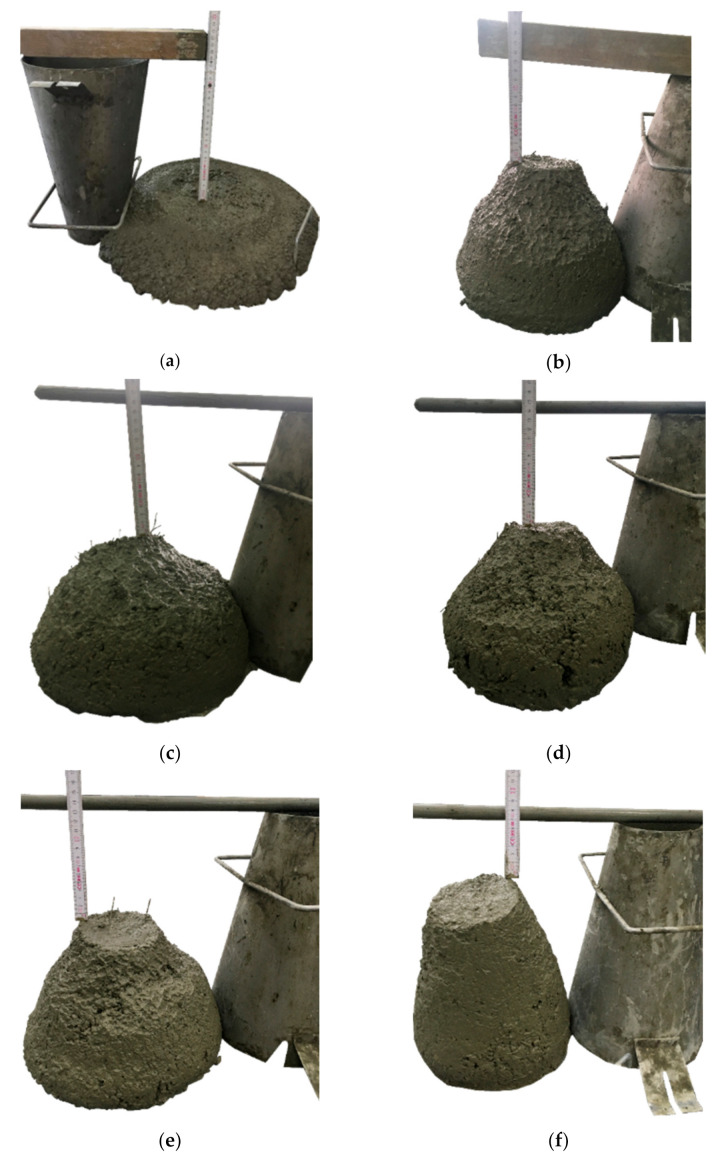
Slump test for concrete mixes: (**a**) PC; (**b**) PM_3; (**c**) PM_2; (**d**) PD_3; (**e**) PD_2; (**f**) FF_2.

All concrete mixes were prepared in the Zyklos planetary rotary mixer by Pemat (Pemat, Freisbach, Germany). Table 4 shows the mixing procedure used in the production of concrete. It is noteworthy that while dosing individual ingredients through a special hole in the mixer, the device was not stopped. After all the materials were mixed, the slump test was carried out according to the standard EN 12350-2 [58] in order to determine the consistency class of fresh concrete. In the beginning, the mold was filled with the first concrete layer and compacted manually by staking (25 times), then the above steps were repeated twice. When the cone was completely filled with the compacted mixture, it was evenly raised. Finally, the slump (h) was measured, which is equal to the difference between the height of the mold and the highest point of the cone. The consistency and slump of the tested concrete mixtures are shown in Figure 1, and the measurement results are presented in Table 5. Additionally, for concrete without fibers, a slump flow test was carried out in accordance with the EN 12350-8 standard [59], which is intended for self-compacting concretes, to check its fluidity. The test consisted of filling the cone with concrete mix and then raising it at a constant speed. When the concrete stopped flowing, two diameters perpendicular to each other were measured. Their length was 560 and 570 mm, thus including PC in the SF1 slump flow class according to EN 206 [60]. In the case of mixtures with fibers, the slump flow tests were not possible to execute because their workabilities were too low. The tests show that the workability of the mixtures was significantly deteriorated due to the addition of synthetic fibers as was observed in different research i.e., [61]. Because of the incorporation of the additional surface to cover, the consistency class decreased by three for FF_2, two for PM_3, PD_3 and PD_2, and one for PM_2 compared to PC. A slight influence of V_f_ was also visible, as in a mixture with 2.0 kg/m^3^ of PM fibers, a greater drop of the cone was recorded than in the same mixture with 3.0 kg/m^3^ of the same fibers. Furthermore, in the case of a mixture with hybrid fibers, the workability was the most severe.

**Figure 2:**
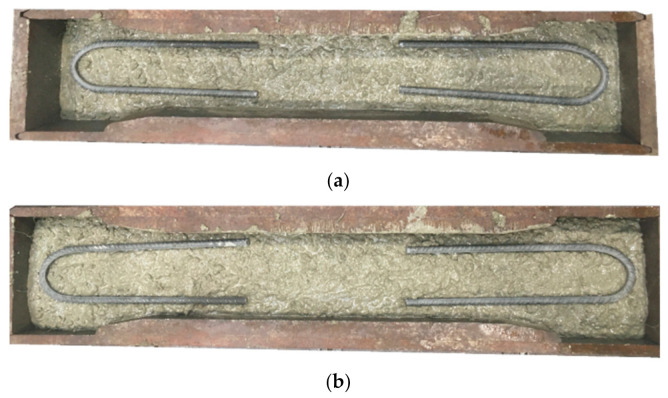
Casting of dog-bone specimens for uniaxial tensile test: (**a**) first layer of stirrups on the 1/3 depth of the sample; (**b**) second layer of stirrups on the 2/3 depth of the sample.

After testing the properties of fresh concrete mixtures, six cubes of dimensions 150 × 150 × 150 mm for compressive strength tests according to EN 206 [60] and six beams of dimensions 150 × 150 × 550 mm^3^ for flexural tensile strength tests in accordance with EN 14651 [28] for each type of concrete were concreted. Additionally, in order to test the tensile strength, one dog-bone specimen was concreted for each series with a height of 750 mm, a middle cross-section of 100 × 100 mm, and a lower/upper cross-section of 100 × 140 mm^2^. It must be noted that dog-bone specimens were specially reinforced by two layers of stirrups on both ends—on the 1/3 depth and 2/3 depth of the sample what is presented in Figure 2. This was done to force the crack occurrence in the middle of the sample. A total of 36 cubes, 18 beams and 6 dog-bone specimens were concreted (Table 6). All samples were stored under a foil and systematically watered to avoid drying and the appearance of shrinkage cracks. After 17 days, the cubes and beams were demolded and left in a room temperature at 20 °C ± 2 °C and ≥95% humidity according to [62] until the test day (36 days for PC; 37 days for PM_3, PM_2, PD_3; 38 days for PD_2, FF_2.). Additionally, on day 18, all beams in the middle of the span were cut-5 mm wide and 25 mm deep along the entire beam width with a diamond saw in accordance with EN 14651 [28].

### 2.2. Methods

**Figure 3:**
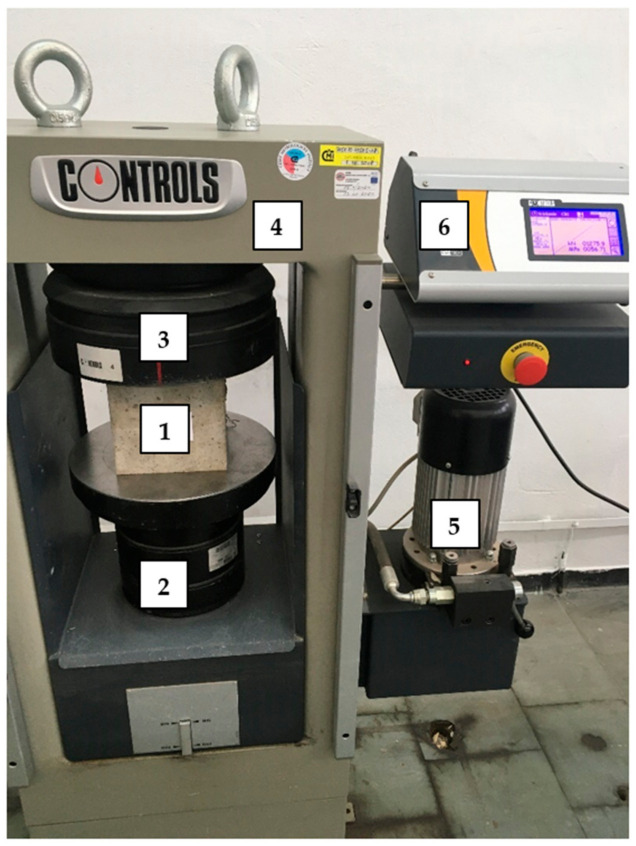
Set-up for compressive strength test: 1—sample; 2—fixed head; 3—movable head; 4—frame; 5—motor; 6—digital display meter.

Regarding the compressive strength test, cubes with dimensions of 150 × 150 × 150 mm^3^ were tested in accordance with EN 206 [60]. The tests were performed on a Controls Model 50-C46CO2 machine (Controls, Liscate, Italy) where the stress was increased at a rate of 0.5 MPa/sec (Figure 3).

**Figure 4:**
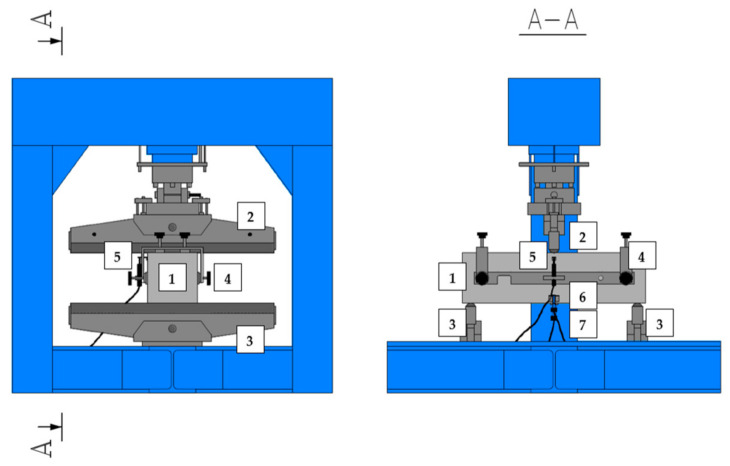
Set-up for three-point bending test: 1—sample; 2—loading roller; 3—supporting roller; 4—rigid frame to install LVDT; 5—LVDT to measure δ; 6—clip gauge to measure CTOD; 7—clip gauge to measure CMOD.

**Figure 5:**
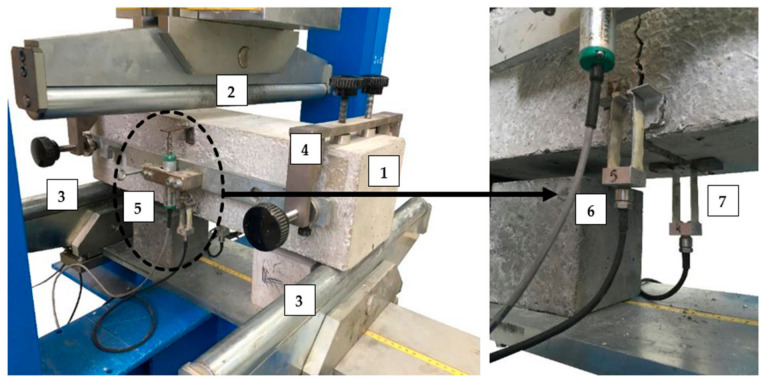
Picture of the sample during the three-point bending test: 1—sample; 2—loading roller; 3—supporting roller; 4—rigid frame to install LVDT; 5—LVDT to measure δ; 6—clip gauge to measure CTOD; 7—clip gauge to measure CMOD.

The presented flexural tensile tests were performed in accordance with the EN 14651 standard [28], which is dedicated to 3PBT for metallic FRC in order to describe their flexural tensile strength. Figure 4 and Figure 5 shows the test set-up with all sensors: linear variable differential transformer (LVDT) for measuring deflection (δ), clip gauges for measuring crack mouth opening displacement (CMOD) and crack tip opening displacement (CTOD), support frame and small steel angles enabling the installation of LVDT. As part of the research, free-supported beams with a cut in the middle and the following dimensions: 150 × 150 × 550 mm^3^ were tested. The span between the supports (l) was equal to 500 mm. The samples were loaded with a force (F) in the middle of the span with a constant increment of δ equal to 0.2 mm/min until reaching δ = 5 mm, i.e., until the end of the test. During the test, three curves were recorded, F-CMOD, F-δ and F-CTOD, though according to EN 14651 [28], the first one is used for the strength characteristics of FRC. Nevertheless, there is a formula (Equation (1)) to calculate the CMOD value using δ.

(1) δ=0.85CMOD+0.04

Thanks to the obtained graphs F-CMOD and F-δ, it was possible to determine the flexural tensile strength in the range of limit of proportionality $f_{\mathrm{ct},L}^{f}$_,_ (Equation (2)) and residual flexural tensile strengths: f_R,1_, f_R,2_, f_R,3_ and f_R,4_ (Equation (3)), using the formulas included in the EN 14651 standard [28]. It must be mentioned that for F-CMOD plot obtained during testing of FRC, CMOD_1_ = 0.5 mm, CMOD_2_ = 1.5 mm, CMOD_3_ = 2.5 mm and CMOD_4_ = 3.5 mm.

(2) $$f_{\mathrm{ct},L}^{f}=\frac{3F_{L}l}{2{\mathrm{bh}}_{\mathrm{sp}}^{2}}$$

(3) $$f_{R,j}=\frac{3F_{j}l}{2{\mathrm{bh}}_{\mathrm{sp}}^{2}}$$

where:

$f_{\mathrm{ct},L\;}^{f}—$ limit of proportionality [N/mm^2^];
$f_{R,j}$—residual flexural tensile strength corresponding to CMOD = CMOD_j_ or δ = δ_j_ (j = 1, 2, 3, 4) [N/mm^2^];
$F_{L}\;$—load corresponding to the limit of proportionality [N];
$F_{j}$—load corresponding to CMOD = CMOD_j_ or δ = δ_j_ (j = 1, 2, 3, 4) [N];
l —span length [mm]—500 mm;
b —width of the specimen [mm]—150 mm;
$h_{\mathrm{sp}\;}$—distance between the tip of the notch and the top of the specimen [mm]—150 mm–25 mm = 125 mm.

**Figure 6:**
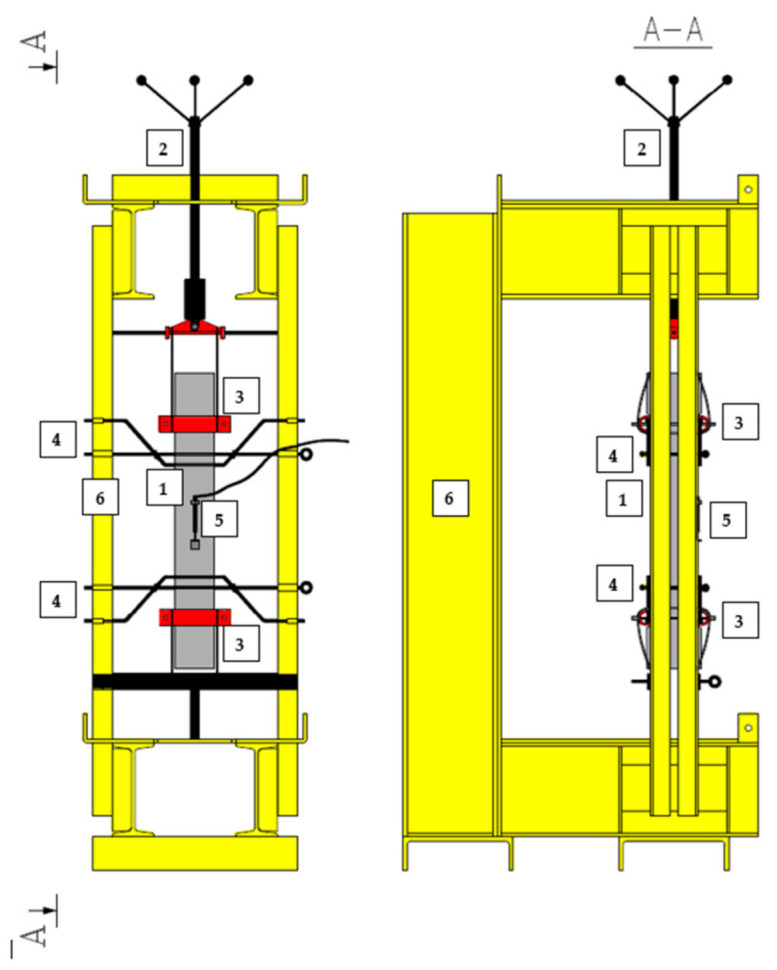
Set-up for uniaxial tensile test: 1-sample; 2—manual load application; 3—grip; 4—bar assembling to prevent falling down of the sample after failure; 5—LVDT to measure w; 6—rigid frame.

**Figure 7:**
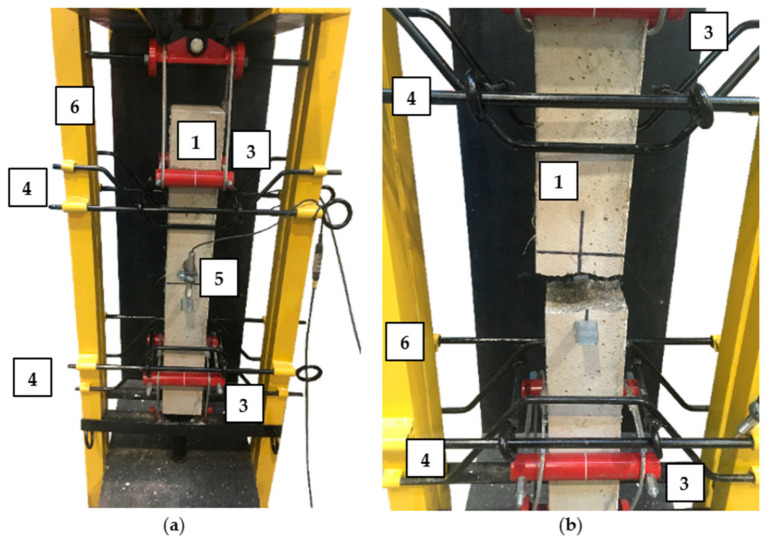
Picture of the sample: (**a**) before the uniaxial tensile test; (**b**) after the uniaxial tensile test: 1—sample; 3—grip; 4—bar assembling to prevent falling down of the sample after failure; 5—LVDT to measure w; 6—rigid frame.

The uniaxial tensile test was performed on larger samples than those usually used in the tests [56,63,64]. In order to execute it, a special set-up was constructed visible in Figure 6 and Figure 7. Red bars were mounted to maintain the sample in the vertical position during the tests and black ones to hold two parts of the specimens after cracking. The tensile force was applied manually from the top of the set-up. Additionally, in the middle of the specimens, from both sides, two LVDTs were installed to measure the extension of the sample assuming that the crack will occur somewhere in the middle, within measured distance of 100 mm. The aim was to obtain the stress-crack opening (σ-w) diagram.

**Figure 8:**
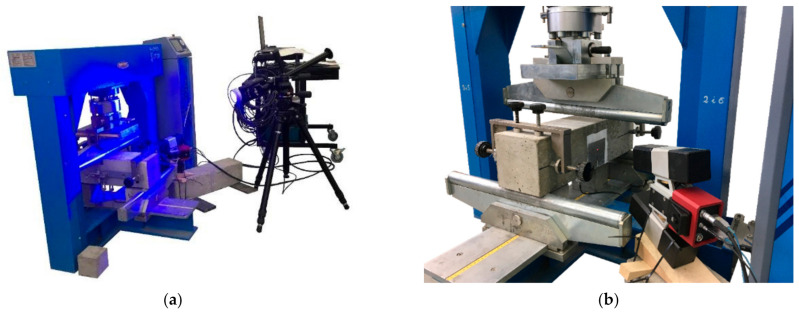
Set-up for monitoring the samples using the DIC system: (**a**) computer for storage the data with the lamp to lightened the studied surface; (**b**) camera.

The Digital Image Correlation technique is based on the idea of comparing the random speckle pattern, which was sprayed on the studied surface, of a reference image with the speckles in deformed images during the test. Thanks to this, it is possible to track the deformations and create the local strain map which allows to characterize both the fracture process zone and the crack. The aim to use the DIC technique was to compare the values of CMOD and CTOD measured in the DIC system with the ones from the clip gauges, and to visualize the crack propagation and to analyze the strain. The set-up of the DIC system is presented in Figure 8. Moreover, the facets had size of 15 × 15 pixels and the images were taken with the frequency 0.5 Hz (1 image every 2 s), and kept in the computer memory for later analysis.

## 3. Results and Discussion

### 3.1. Compressive Strength

For each concrete mix, six cubes with dimensions of 150 × 150 × 150 mm^3^ were tested for compressive strength (f_c_) in accordance with EN 206 [60]. The tests were performed on a Controls Model 50-C46CO2 machine (Controls, Liscate, Italy) where the stress was increased at a rate of 0.5 MPa/sec. The mean f_cm_, standard deviation s_fc_, and the coefficient of variation V_fc_ of the compressive strength for PC, PM_3, PM_2, PD_3, PD_2, and FF_2 are summarized in Table 7. It should be noted that samples no. 5 for PC and PD_2 and sample no. 6 for PM_3 were rejected during the analysis, as they significantly differed from the other results. This could be due to inaccurate mixing of the concrete components at the bottom of the mixer, as the samples were concreted as one of the last in the series.

**Figure 9:**
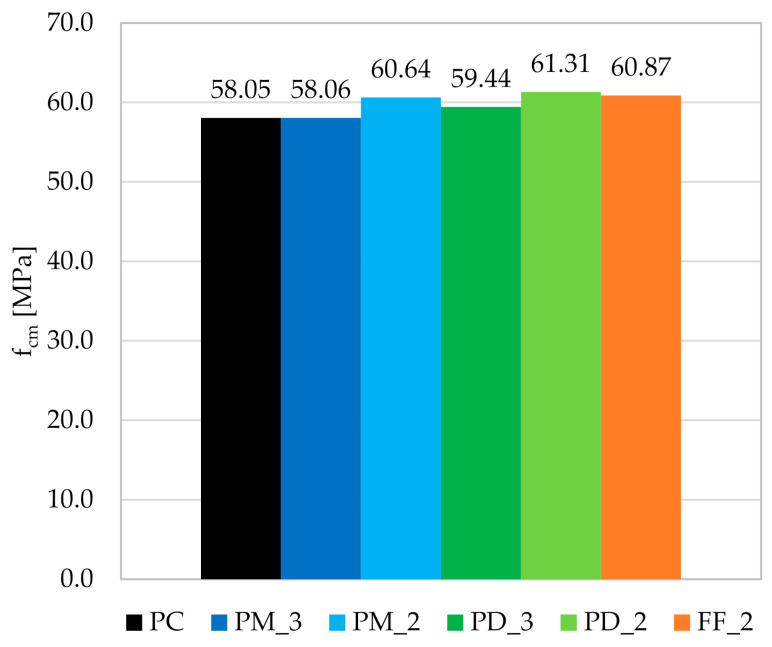
Graph of the mean compressive strength f_cm_ for individual concrete mixes.

The addition of fibers to the concrete mix increased the f_cm_ of the PM_2; PD_3; PD_2, and FF_2 by respectively 4.5; 2.4; 5.6, and 4.9% and did not affect the f_cm_ of the PM_3 series when comparing with the PC (Figure 9). Moreover, it can be seen that the reduction of V_f_ from 3 kg/m^3^ to 2 kg/m^3^ had a positive effect on f_cm_. On the other hand, the means of individual FRC mixes were from 58.05 to 61.31 MPa, standard deviations from 0.72 to 2.77 MPa, and the coefficients of variation from 1.19 to 4.66%, which showed that the variation between the series was not very large. The conclusion can be made that the type and the amount of fibers did not significantly affect the f_cm_, which is in agreement with many other studies, e.g., [65,66,67]. It is also worth noting that the clear deterioration of the workability of the concrete did not have a negative effect on the f_c_. Additionally, the 5% quantile of the concrete compressive strength (f_c;0.05_) was calculated, which means the probability of occurring the value of f_c_ less than 0.05. For the distribution of t-non-centrifugal with a one-sided rejection region, the quantile can be determined from Equation (4). The results of the f_c;0.05_ calculations are presented in Table 7.

(4) $$f_{c;0.05}=f_{\mathrm{cm}}-t_{n-1,\alpha }\frac{s_{\mathrm{fc}}}{\sqrt{n}}$$

where:

n—number of samples (n = 6 or 5) [-];
s_fc_—standard deviation of compressive strength [MPa];
t_n-1,α_—with a confidence level of α = 0.05, for six samples (n − 1 = 5) − t_5;0.05_ = 1.67, for five samples (n − 1 = 4) − t_4;0.05_ = 1.46 [-].

### 3.2. Flexural Tensile Strength

**Figure 10:**
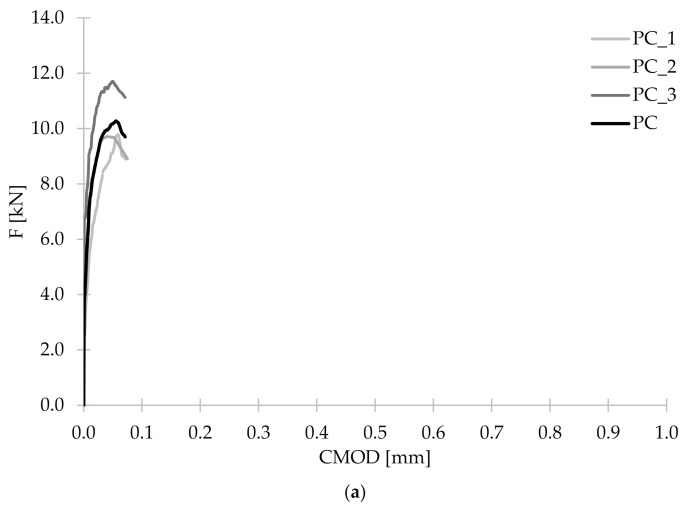

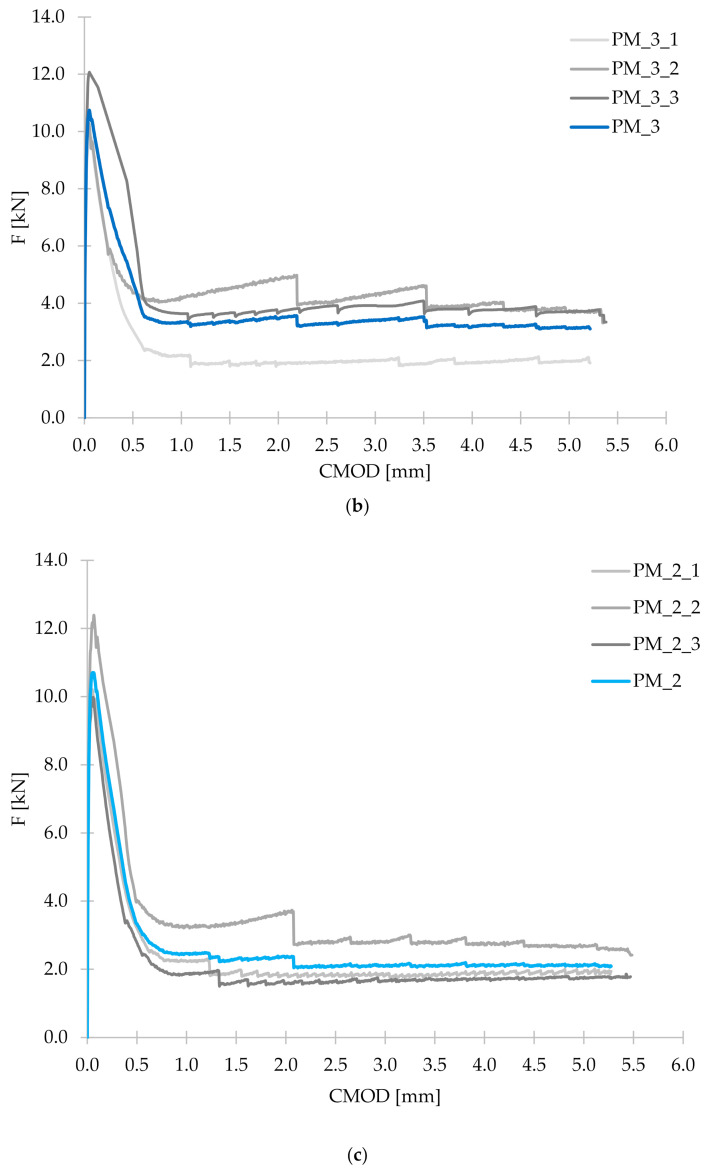

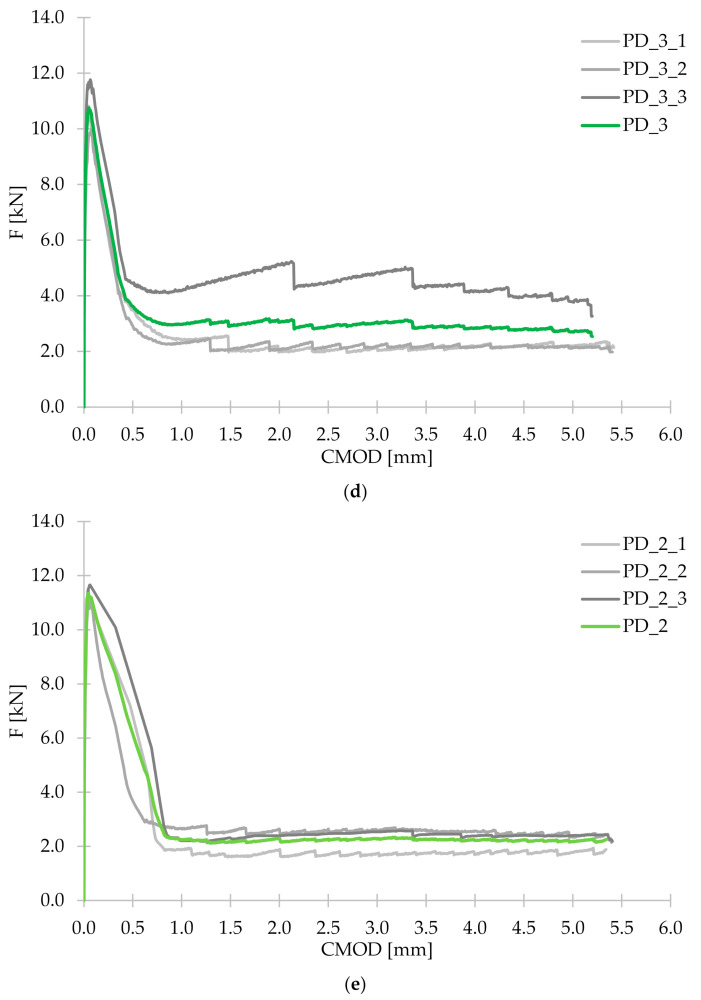

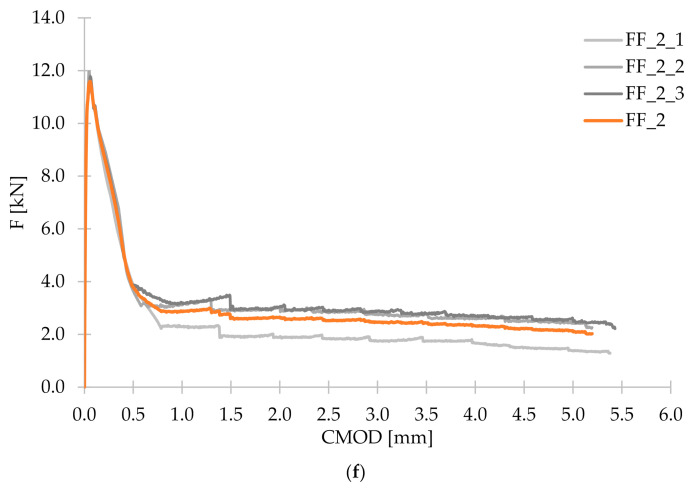
Graphs of mean F-CMOD curves for (**a**) PC; (**b**) PM_3; (**c**) PM_2; (**d**) PD_3; (**e**) PD_2; (**f**) FF_2 with graphs of the individual samples within the series.

**Figure 11:**
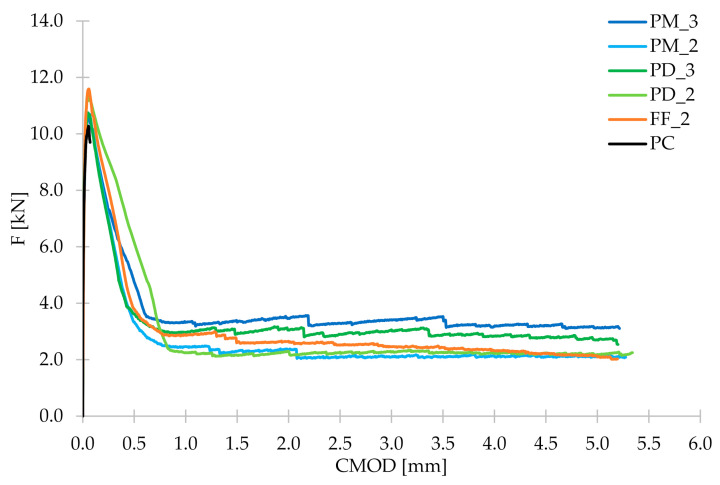
Graphs of mean F-CMOD curves for all concrete mixes.

**Figure 12:**
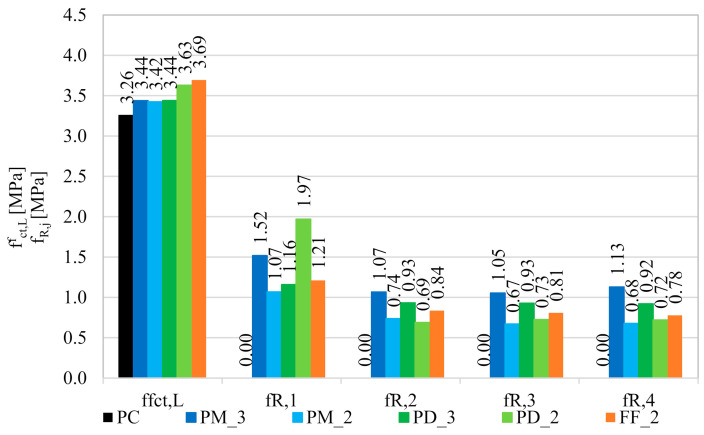
Comparison of the mean values of the flexural tensile strength in the range of limit of proportionality ($f_{\mathrm{ct},L}^{f}$) and residual flexural tensile strengths (f_R,j_) for the tested concrete mixtures.

Within a three-point bending tests according to EN 14651 [28], three beams were examined for each of the concrete mixes. As a result of the measurement of F and CMOD, the F-CMOD graphs presented in Figure 10 were made for each mixture. All plots of the averaged F-CMOD curves for each series are visible in Figure 11. In the beginning, in accordance with the test methodology, the loads corresponding to the maximum load for CMOD ≤ 0.05 mm (F_L_) and to CMOD = 0.5 (F_1_); 1.5 (F_2_); 2.5 (F_3_), and 3.5 mm (F_4_) were determined for the tested concrete mixes (Table 8). Subsequently, the flexural tensile strength in the range of limit of proportionality ($f_{\mathrm{ct},L}^{f}$) and residual strengths: f_R,1_; f_R,2_; f_R,3_; f_R,4_ were calculated and are shown in Figure 12.

As a result of the addition of synthetic fibers to concrete, $f_{\mathrm{ct},L}^{f}$ increased approximately by 5.5% for PM_3, PM_2, and PD_3; by 11.5% for PD_2 and by 13.5% for FF_2. In conclusion, the smallest improvement was noted in the case of a mixture with one type of fibers, and higher l_f_. Mixes with slightly shorter fibers (l_f_ = 48 mm) performed better, in particular where fewer fibers (2 kg/m^3^) have been used. However, the best results were obtained for a hybrid blend with V_f_ = 2 kg/m^3^. This may be because of the fact that the combination of two types of fibers more effectively bridged the cracks that appear in the initial stage of cracking, and the lower fiber content allowed for more even distribution of all fibers in the concrete. Furthermore, when comparing the results with the ones obtained in the other literature positions, it occurred that the level of enhancement in the flexural tensile strength (f_fl_) is in agreement with many of them. For example in [68], where V_f_ was equal to 0.33%, fibers were 60 mm long and with a diameter of 1.0 mm, f_fl_ increased by 6.24%. Additionally, Meza et al. reported in [69] that the addition of 2.7 kg/m^3^ of copolymer (l_f_/d_f_ = 38 mm/2.0 × 0.5 mm) fibers resulted in 6.44% increase of f_fl_. Finally, Rucka et al. in [40] tested exactly the same type of fibers PM with the dosage equaled to 2 kg/m^3^ what resulted in a 6.83% f_fl_ increase. There also exist studies in which the improvement in strength was smaller, even though fibers with similar length and content were used, namely f_fl_, which increased from 2.96 to 4.41%, depending on the V_f_ in [70]. In the other case, comparable enhancement was obtained for almost twice higher amount of fibers, like in [71] where after the addition of 0.50% of polypropylene fibers (l_f_/d_f_ = 38 mm/0.91 mm) f_fl_ increased from 3.25 to 3.70 MPa, so by 13.8%. One should also take into account the fact that in many studies the decrease of f_fl_ was noted despite the addition of fibers, like in [72,73,74].

**Figure 13:**
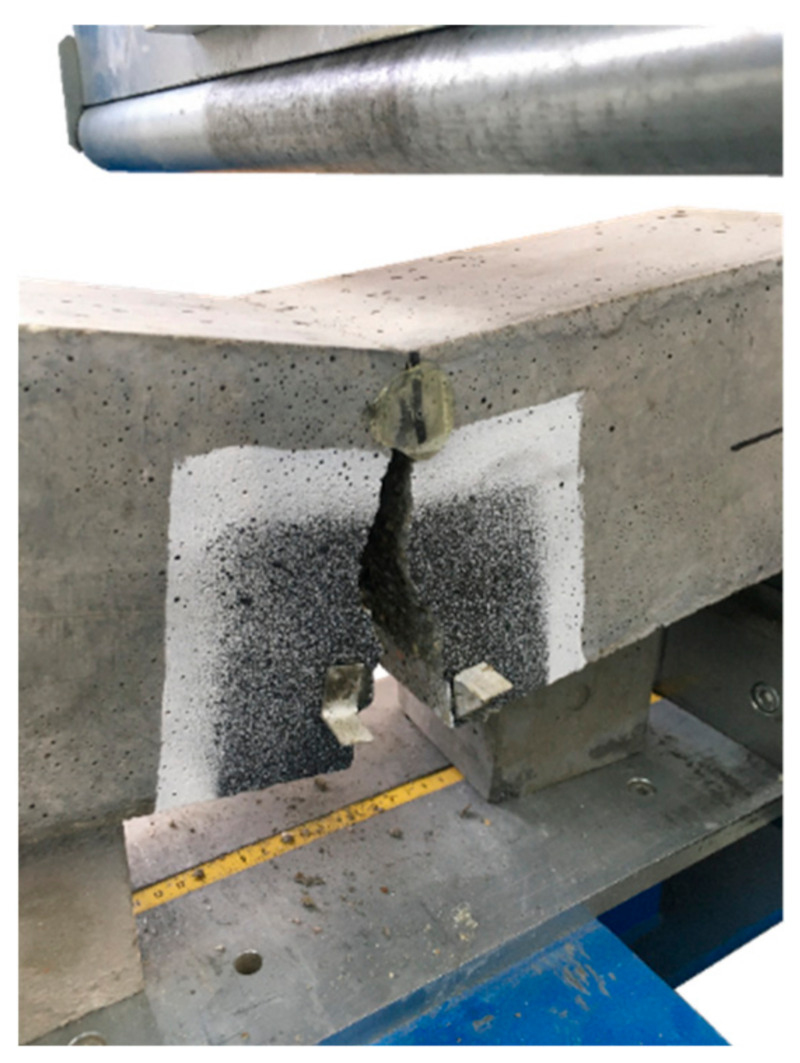
PC_1 beam without fibers after the three-point bending test.

**Figure 14:**
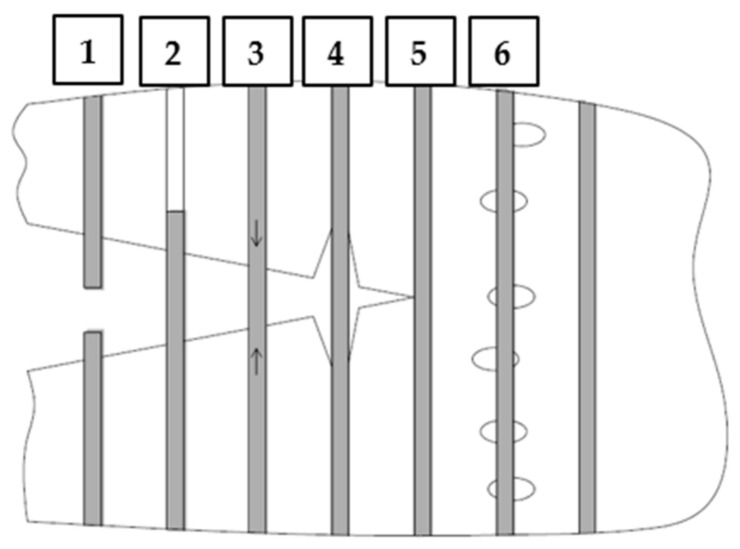
Failure mechanisms of FRC: 1—fiber rapture; 2—fiber pull-out; 3—fiber bridging the macrocrack; 4—matrix/fiber debonding; 5—fiber counteracting crack propagation; 6—fiber bridging the microcracks.

In the case of plain concrete, there was a sudden, brittle failure after cracking and it was impossible to determine its residual strengths. Figure 13 presents the sample PC_1 immediately after the bending test, which caused it to split in two. On the other hand, FRC beams, after a decrease in the transferred force, were still able to resist significant load with increasing CMOD. Therefore, in the non-elastic range, an improvement in ductility is seen after the addition of fibers. From the comparison of the residual strengths for different FRC mixes it can be concluded that usually f_R,j_ reached higher values when the fiber content was higher, so V_f_ = 0.33% (Figure 11 and Figure 12). Moreover, after cracking, the PM_3 beam behaved best, wherein higher V_f_ and longer fibers with l_f_ = 54 mm were added, so those that will more efficiently bridge the macrocracks appearing in the second phase of cracking. It is also noteworthy that among the mixtures with 2 kg/m^3^ the highest f_R,2_; f_R,3_; and f_R,4_ are achieved for FF_2 series, which may again indicate the positive effect of combining two types of fibers. The graphs of F-CMOD for FRC (Figure 10) also show some vertical jumps, which were the result of fibers’ failure in the crack cross-section. Failure mechanisms of concrete with fibers are presented and described in Figure 14. Namely, it can be said that in an uncracked concrete the fibers are inactive, however when micro-and macro-cracks occur (6 and 3, respectively), fibers start to bridge them and transfer tensile stresses [75]. Additionally, fibers can prevent further propagation of the crack’s tip (5). Later on, it can happen that fibers will debond from the matrix (4); pull-out (2), or rupture (1), and it will lead to absorption and dissipation of some energy, as a result, stabilization of crack growth in the concrete [75]. It must be noted that in the current study, the rupture of fibers was observed. Often, after breaking the fiber, there was a slight strengthening of the concrete, as the force that it was able to transfer increased with increasing CMOD. It was visible during the analysis of f_R,j_ for: PM_3, where f_R,4_ > f_R,3_; PM_2, where f_R,4_ > f_R,3_; PD_2, where f_R,3_ > f_R,2_ and in Figure 11 and Figure 12. It should also be mentioned that the results for individual samples within the series indicated a significant dispersion of the F-CMOD curves, which was a result of a small fracture area, and thus a large statistical variability of the amount of fibers crossing this surface [76]. Therefore, when testing according to EN 14651 [28], a larger number of samples should be studied. Nevertheless, thanks to this method, which is intended for testing concretes with metallic fibers, a smaller scatter of results is obtained for samples with synthetic fibers than for steel ones [10,76,77]. This can be a result of a more homogeneous distribution of non-metallic fibers than metallic fibers in the concrete mix. It is also worth paying attention to the fact that after the end of the test the FRC beams did not split in half, but retained their integrity. Moreover, all the beams were damaged by a quasi-vertical crack beginning in the sample notch.

#### 3.2.1. Empirical Equations for Flexural Tensile Strength

In the literature, some formulas for the calculation of f_fl_ of FRC can be found. Legeron and Paultre [42] suggested that f_fl_ may be calculated by Equation (5) using the characteristic compressive strength f_ck_ of steel FRC and the coefficient λ being in the range of 0.35–0.65, but usually, the value of 0.5 is assumed. This formula raises doubts, however, as it does not take into account the material, geometry and volume content of the fibers, which are used in the concrete mix, and only the f_ck_ itself. Another suggestion is Equation (6) presented by Glinicki in [43,44], also for FRC with steel fibers, which takes into account both the nominal fiber content (V_f_) and the fiber slenderness (l_f_/d_f_). On the other hand, Swamy and Mangat [45] presented Equation (7), which not only makes f_fl_ dependent on the properties of the metallic fiber, but also on the flexural tensile strength of concrete without fibers (f_flc_).

(5) $$f_{\mathrm{fl}}=\lambda \sqrt[{3}]{f_{\mathrm{ck}}{}^{2}}$$

(6) $$f_{\mathrm{fl}}=0.73+8.061V_{f}\frac{l_{f}}{d_{f}}$$

(7) $$f_{\mathrm{fl}}=0.97\left(1-V_{f}\right)f_{\mathrm{flc}}+3.41V_{f}\frac{l_{f}}{d_{f}}$$

As can be seen, all presented formulas are given for the calculation of f_fl_ for concrete with metallic fibers. Therefore, it was checked whether their use in the case of the tested concretes with synthetic fibers would be justified, and the results of the calculations are presented in Table 9, where $f_{\mathrm{ct},L}^{f}$ = f_fl_.

**Figure 15:**
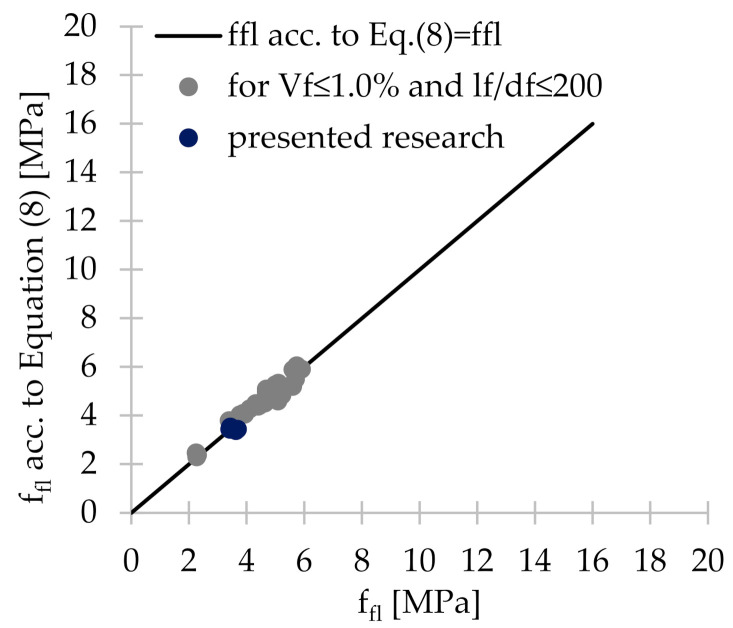
Graphical representation of the correlation of presented research and selected literature positions with the empirical formula for the flexural tensile strength f_fl_ proposed by the authors.

From Table 9 it can be concluded that Equation (5) dependent only on f_ck_ gave results more than twice higher than those obtained in the current study. Better correlation is achieved when Equation (6) was used because the obtained results were lower by 17–41%, except for PM_3 for which they were 14% higher than those obtained in 3PBT. On the other hand, the best agreement was obtained when Equation (7) was applied since the values of f_fl_ were 1.03–1.31 times greater than f_fl_ from the performed tests. Given the fact that steel fibers have a greater influence on the mechanical properties of concrete than synthetic fibers [9,10,69] Equation (7) was adjusted in such a way that the concrete matrix would have a greater and the added fibers smaller influence on the calculated f_fl_. Therefore, the authors proposed Equation (8). Table 10 compares the results obtained for the conducted research and the results included in selected literature positions with the results obtained for the empirical formula for flexural tensile strength f_fl_ proposed by the authors. It can be concluded that Equation (8) correlated very well with the results obtained during the PM_3; PM_2; PD_3; PD_2; and FF_2 tests, as the obtained values were respectively 2.4%; 0.2% greater and 0.3%; 7.1%; 7.3% lower than $f_{\mathrm{ct},L}^{f}$ = f_fl_. As was mentioned before, the validity of using Equation (8) was checked, when synthetic fibers of a different length, diameter, volume content and concrete mixes of various compositions included in other literature references were used. The analysis showed that the fiber content in the concrete has a significant influence on the degree of correlation. Namely, for V_f_ ≤ 0.5%, there was usually quite a good agreement with the conducted research (f_fl_ acc. to Equation (8)/f_fl_ = 0.967–1.084). Moreover, when 0.5% ≤ V_f_ ≤ 1.0%, then f_fl_ was from 7.2% lower to 11. 3% greater than the result obtained from the performed tests. In case of higher V_f_ the correlation was rather poor. Therefore, it can be concluded that Equation (8) can be successfully used to estimate the value of f_fl_ when V_f_ ≤ 1.0%. It is noteworthy that also the fiber geometry had a significant influence on the level of correlation with the proposed formula. Fibers with l_f_/d_f_ greater than 200, i.e., equal to 480 and 600, gave values respectively 1.17–2.55 and 1.23–1.42 times greater than the ones from the tests depending on the V_f_. To sum up, Equation (8) can be profitably used when the slenderness of the polymer fibers is less than 200 and V_f_ ≤ 1.0% (Figure 15).

(8) $$f_{\mathrm{fl}}=1.00\left(1-V_{f}\right)f_{\mathrm{flc}}+0.70V_{f}\frac{l_{f}}{d_{f}}$$

#### 3.2.2. Fiber-Reinforced Concrete Classification

Model Code 2010 [26] proposes a method of FRC classification based on the three-point bending test carried out in accordance with the EN 14651 standard [28]. It uses the characteristic values of the limit of proportionality and residual flexural tensile strengths corresponding to CMOD = 0.5 and 2.5 mm: $f_{\mathrm{ct},\mathrm{Lk}}^{f}$, f_R,1k_, and f_R,3k_, respectively. These values are calculated from Equation (9) and Equation (10). It is worth mentioning that f_R,1k_ corresponds to the serviceability limit state (SLS), and f_R,3k_ corresponds to the ultimate limit state (ULS). The rules for classifying Parameters 1 and 2 and those regarding the possibility of total or partial replacement of traditional reinforcement in a form of steel bars by fibers are described in Table 11.

(9) $$f_{\mathrm{ct},\mathrm{Lk}}^{f}=f_{\mathrm{ct},\mathrm{Lm}}^{f}-k\cdot s_{\mathrm{ct},L}^{f}$$

(10) $$f_{R,\mathrm{jk}}=f_{R,\mathrm{jm}}-k\cdot s_{R,j}$$

where:

$f_{\mathrm{ct},\mathrm{Lk}}^{f}$—characteristic limit of proportionality [N/mm^2^],
$f_{\mathrm{ct},\mathrm{Lm}}^{f}$—mean limit of proportionality [N/mm^2^],
f_R,jk_—characteristic residual flexural tensile strength corresponding to CMOD = CMOD_j_ or δ = δ_j_ (j = 1,2,3,4) [N/mm^2^],
f_R,jm_—mean residual flexural tensile strength corresponding to CMOD = CMOD_j_ or δ = δ_j_ (j = 1,2,3,4) [N/mm^2^],
$S_{\mathrm{ct},L}^{f}$—standard deviation of $f_{\mathrm{ct},L}^{f}$ from $f_{\mathrm{ct},\mathrm{Lm}}^{f}$ [N/mm^2^] calculated from Equation (11);
s_R,j_—standard deviation of f_R,j_ from f_R,jm_ [N/mm^2^] calculated from Equation (12)
  (11) $$s_{\mathrm{ct},L}^{f}=\sqrt{\frac{\sum {\left(f_{\mathrm{ct},\mathrm{Lm}}^{f}-f_{\mathrm{ct},L}^{f}\right)}^{2}}{\left(n-1\right)}}$$
  (12) $$s_{R,j}=\sqrt{\frac{\sum {\left(f_{R,\mathrm{jm}}-f_{R,j}\right)}^{2}}{\left(n-1\right)}}$$
  where:
  n—number of samples [-],
  k—factor depending on the number of samples given in Table 2 in Model Code 2010 [26], for 3 samples it is equal to 1.89.

Table 12 presents the results of the FRC classification according to Model Code 2010 [26]. Firstly, only for FF_2 it was possible to assign the mix to Parameter 1 which was equal to 1.0. For the remaining series, all values of f_R,1k_ were smaller than 1.0. Additionally, only for the mixtures PM_3 and PD_2 the ratio f_R,3k_/f_R,1k_ was greater than 0.5, so it is possible to assign them to letters *c* and *a* of Parameter 2, respectively. It is also not possible to completely or partially replace the traditional reinforcement with the fibers, as none of the concrete met the two above-mentioned requirements simultaneously: f_R,1k_/$f_{\mathrm{ct},\mathrm{Lk}}^{f}$ > 0.4 and f_R,3k_/f_R,1k_ > 0.5. It is also worth mentioning that the characteristic values of the residual strengths were significantly reduced as a result of the large standard deviation ($S_{\mathrm{ct},L}^{f}$ and s_R,j_) between samples in a given series. In future tests, it is advisable to increase the number of samples within each mixture. Furthermore, concrete mixes with different amounts of cement, coarse and fine aggregate, water and superplasticizer should be tested to verify made conclusions.

### 3.3. Fracture Energy and Toughness

**Figure 16:**
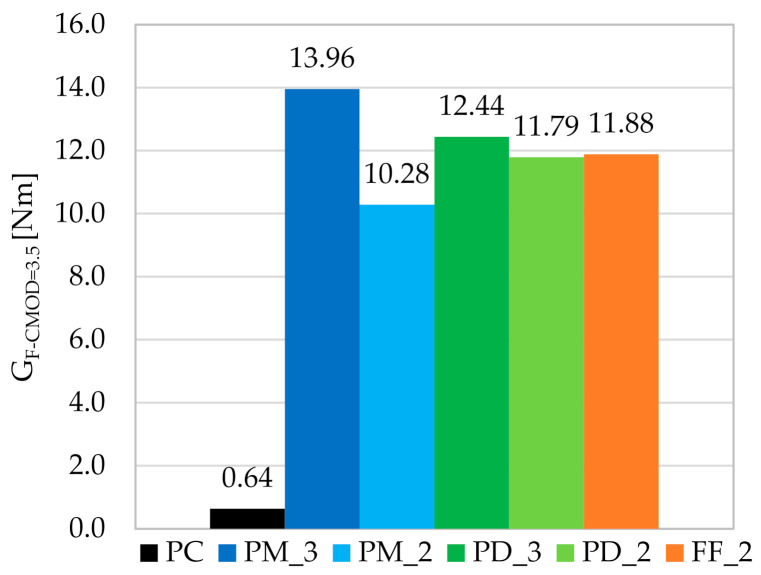
The mean fracture energy calculated up to CMOD = 3.5 mm for individual concrete mixes.

The area under the F-CMOD curve is called the fracture energy-toughness (G_F-CMOD_). In the article, this area is counted up to CMOD = 3.5 mm. Figure 16 shows a graph of the mean fracture energies (G_F-CMOD=3.5_) calculated for all three specimens within each mixture. It can be concluded that specimens with more fibers performed best. The calculated toughness index T_i_, which is equal to the ratio of G_F-CMOD=3.5_ for FRC to G_F-CMOD=3.5_ for PC samples, indicates that the fracture energy for PM_3 and PD_3 increased 22 and 19.6 times, respectively (Table 13). On the other hand, among the mixtures with V_f_ = 2kg/m^3^, the best results were achieved for samples FF_2, where a hybrid blend was used. Compared to other studies in the literature such as [76,77], for similar or higher macrofibers contents in the mix, the ratio of G_F-CMOD_ of plain concrete to G_F-CMOD_ of FRC was much lower. There are also studies [80] where microfibers with V_f_ = 0.50% were used, and the G_F-CMOD_ value was two times lower than the fracture energy presented in this article, despite the higher fiber content. However, it is related to the fact that shorter fibers have a greater influence when concrete is in the initial stage of flexural tensile test, so up to the point of crack formation. It is because they more effectively bridge the smaller cracks and their amount in the concrete mixture is higher. Moreover, in [71] for lightweight concretes with the addition of slightly shorter polypropylene fibers with l_f_/d_f_ = 38 mm/0.91 mm but with higher V_f_ = 0.5; 0.7; 0.9; 1.1; 1.3% similar values of G_F-CMOD_ = 13.88; 16.60; 18; 13; 23.26; 18.27 Nm, respectively, were obtained. All this indicates the logicality of the obtained results for the PC, PM_3, PM_2, PD_3, PD_2 and FF_2.

**Figure 17:**
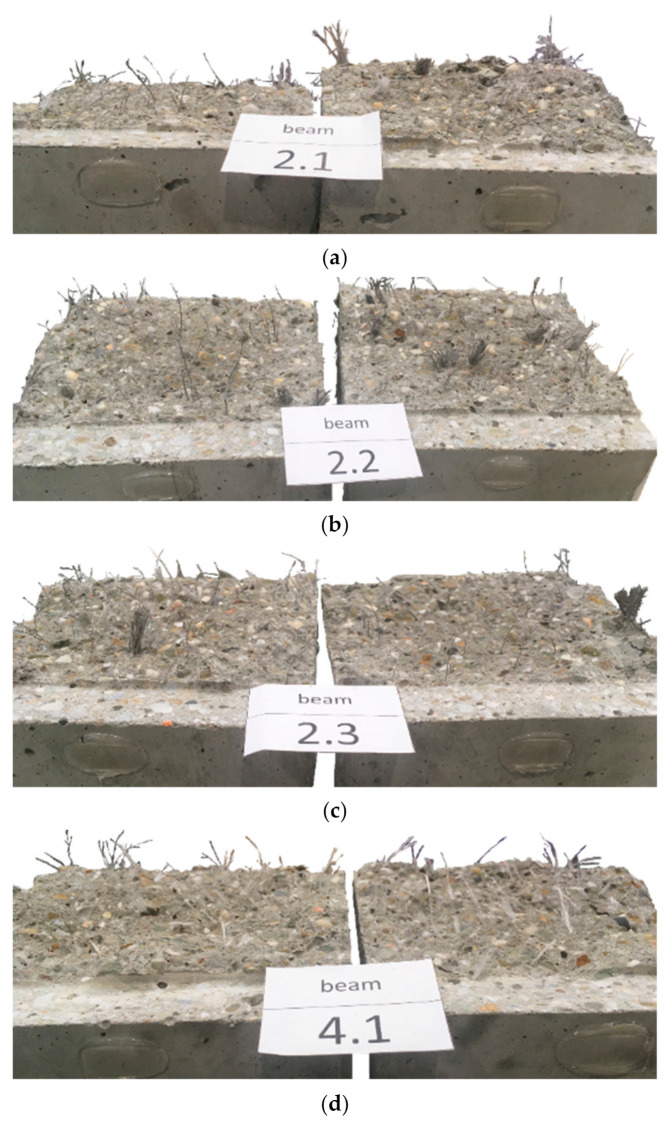

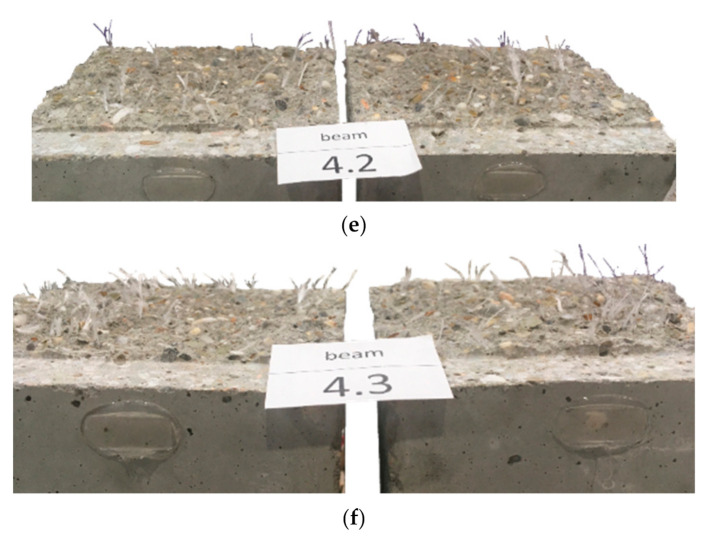
FRC beams broken in the crack cross-section: (**a**) PM_3_1; (**b**) PM_3_2; (**c**) PM_3_3; (**d**) PD_3_1; (**e**) PD_3_2; (**f**) PD_3_3.

A significant increase in the level of fracture energy absorption indicates the positive effect of synthetic fibers on the ductility of concrete, which makes it possible to avoid sudden, brittle failure of the FRC element. In addition, the greater the fiber content, the greater the toughness. Table 13 shows the standard deviation (s_GF-CMOD=3.5_) and the coefficient of variation (V_GF-CMOD=3.5_) of the fracture energy. All the mixtures were characterized by quite high s_GF-CMOD=3.5_ and V_GF-CMOD=3.5_, which is also visible in Figure 10. In order to investigate the cause of such a large discrepancy between the samples, each beam was broken and photographed in the crack cross-section. Figure 17 presents three samples of the mixture PM_3 (PM_3_1, PM_3_2, PM_3_3) and PD_3 (PD_3_1, PD_3_2, PD_3_3) with the largest s_GF-CMOD=3.5_. In the case of the PM_3 mix, it was noticeable that some of the twisted fibers did not disintegrate, which influenced the distribution of the fibers in the crack cross-section and make it uneven. In order to avoid this situation in the future, the fibers should be added at the initial stage along with aggregates so that they have the possibility of breaking the bundles of fibers. A similar situation occurred in the case of PM_2. On the other hand, in the PD_3 mixture, there was no tendency to create bundles of fibers and the PD_2 mixture was characterized by the lowest V_GF-CMOD=3.5_. Thus, the reason for such large differences in G_F-CMOD=3.5_ in PD_3 may be connected with a significantly different number of fibers in the crack cross-section. It is noteworthy that the F-CMOD plot for the sample PD_3_3 clearly differed from F-CMOD plots for PD_3_1 and PD_3_2 and took much larger force values for the same values of CMOD. Additionally, by comparing Figure 17d–f it can be seen that the latter shows a greater amount of fibers in the failure cross-section. In order to verify this conclusion more precisely, it would be necessary to count the fibers in the crack cross-section for all PD_3 samples and compare them.

### 3.4. Tensile Strength

**Figure 18:**
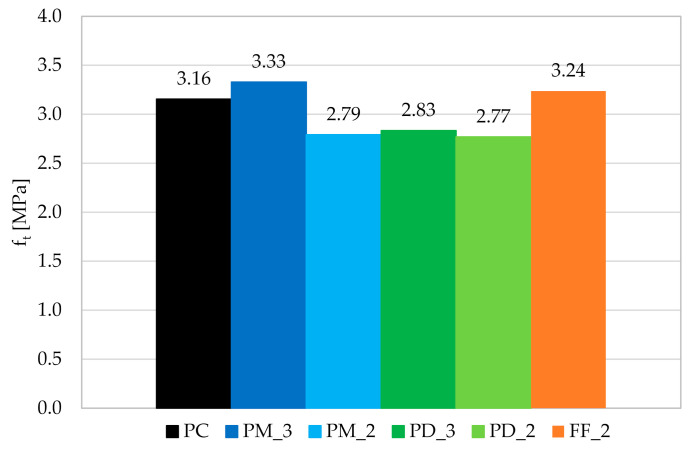
Graph of the mean tensile strength for individual concrete mixes.

The results of the maximum tensile strength f_t_ obtained during the UTT are presented in Figure 18. It can be concluded that the addition of fibers did not have a clear influence on f_t_. Despite PM_3 and FF_2, the samples had lower strength by around 11.5%. On the other hand, the small effect of V_f_ is visible, since mixes with 3 kg/m^3^ behaved better than the ones with 2 kg/m^3^ of the same fibers. Additionally, the hybrid mixture had f_t_ 2.6% higher than the mix with no fibers. As was seen before, FF_2 performed usually better than other concrete mixtures with 2 kg/m^3^ of fibers. In future research, a higher number of tested samples should be used to limit the influence of different amounts of fibers in the cracked cross-section within one mixture.

**Figure 19:**
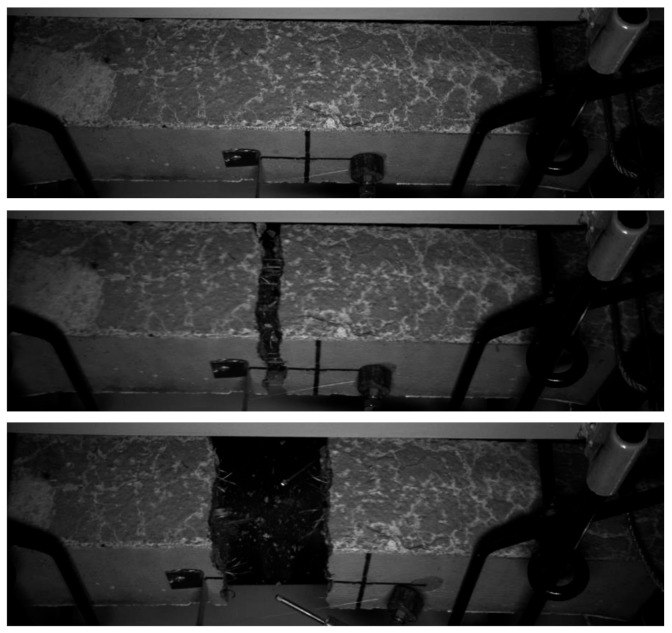
Cracking of PM_2 dog-bone specimen during the tensile test.

In the case of plain concrete, it was not expected to obtain σ-w curve since brittle failure was predicted. On the other hand, studied FRC were supposed to behave in a more ductile way. However, for none of them σ-w curve was received because the failure was really sudden. In Figure 19, cracking of PM_2 dog-bone specimen can be seen during a tensile test, where the time difference between taking pictures was equal to less than 0.02 sec. Moreover, it must be mentioned that four from six samples (PM_3; PD_3; PD_2; FF_2) cracked in the place out of measured range, where the stirrups ended. The reason for not obtaining the σ–w curve may be connected with a relatively small amount of fibers and/or flaws of the set-up, for example manual application of tensile force without a constant increase.

### 3.5. CMOD-δ, CMOD-CTOD and CTOD-δ

**Figure 20:**
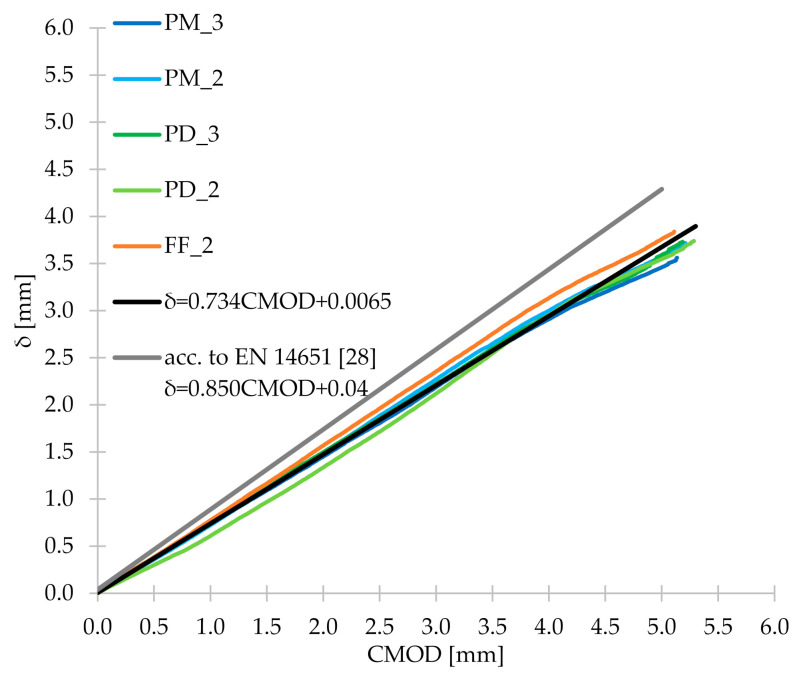
CMOD-δ diagram for the tested concrete mixes with the formula described in EN 14651 [28] and Equation (13) proposed by the authors.

**Figure 21:**
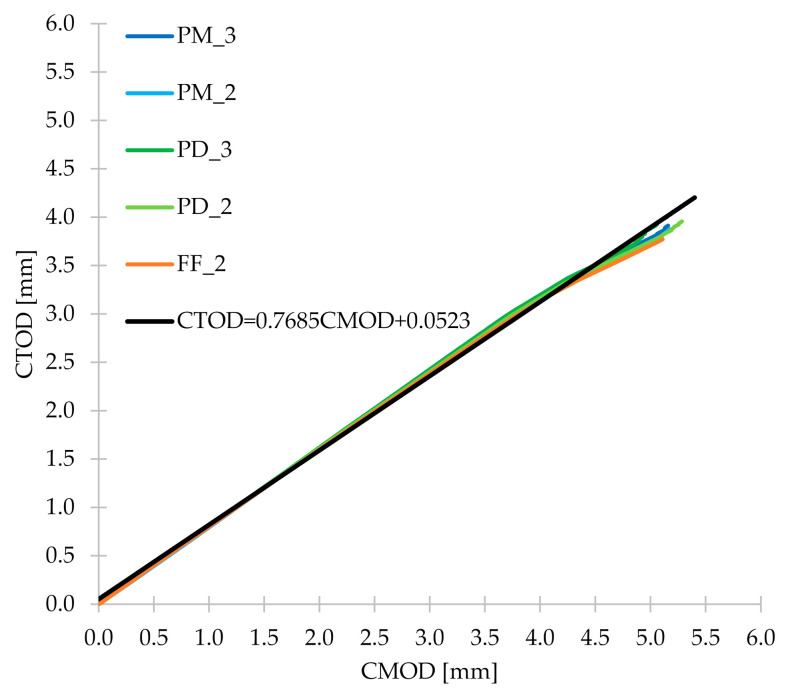
CMOD-CTOD diagram for the tested concrete mixes together with Equation (14) proposed by the authors.

**Figure 22:**
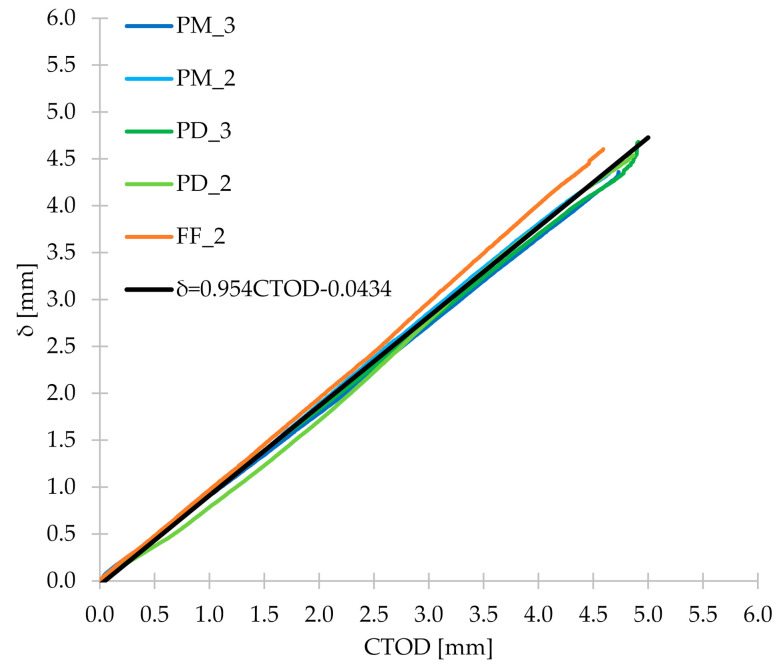
CTOD-δ diagram for the tested concrete mixes together with Equation (15) proposed by the authors.

During the three-point bending test, apart from CMOD measurements, the corresponding CTOD and δ were also measured. Thanks to this, it was possible to determine the relationship between them and Figure 20 shows the first one: CMOD-δ. From Figure 20, it can be concluded that for all concrete mixes tested in the research, the CMOD-δ plots were very similar. Therefore, the mean of the five FRC series was calculated, and then Equation (13) was determined, which correlated very well with this mean (R^2^ = 0.999). Figure 20 also shows the line representing Equation (1), which describes the relationship between CMOD and δ contained in the EN 14651 standard [28]. As can be seen, it differs from the lines determined for FRC mixtures and correlates with them worse than Equation (13) proposed by the authors. Then, in Figure 21, CMOD-CTOD plots are visible, which practically overlap each other. As in the case of CMOD-δ, the formula of the line describing the mean CMOD-CTOD (R^2^ = 0.998) was determined (Equation (14)). For the CTOD-δ relationship (Figure 22) the procedure algorithm was the same as for CMOD-CTOD and the formula proposed by the authors is marked as Equation (15). Equations (13)–(15) can be used to calculate δ, CMOD and/or CTOD when only one of the values is measured during the three-point bending test. Additionally, it is worth noting that all the discussed relationships are linear and directly proportional.

(13) δ=0.734CMOD+0.0065

(14) CTOD=0.7685CMOD+0.0523

(15) δ=0.954CTOD−0.0434

### 3.6. Digital Image Correlation

**Figure 23:**
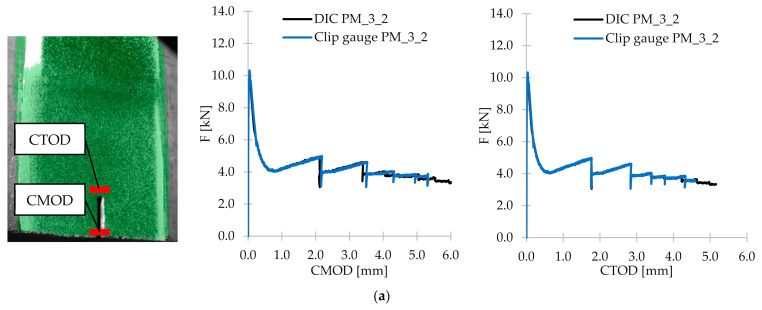

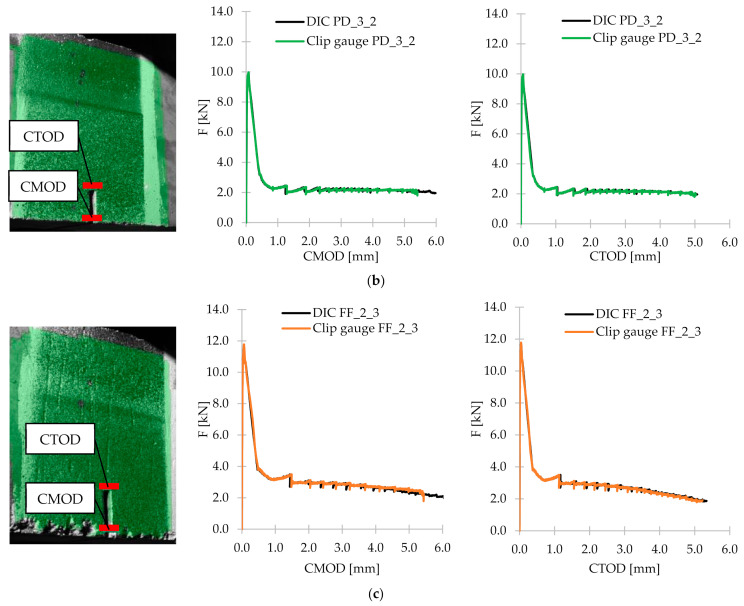
Comparison of CMOD and CTOD results from clip gauges and the DIC system for selected samples: (**a**) PM_3_2; (**b**) PD_3_2; (**c**) FF_2_3.

The purpose of carrying out the DIC technique was to compare the values obtained from clip gauges with CMOD and CTOD measurements from optical system and to visualize the crack propagation and to analyze the strains. Firstly, all 15 specimens (only FRC samples) were subjected to the DIC post-processing analyzes in the GOM Correlate 2020 software. Then, the CMOD and CTOD values were read from the program and together with the results from clip gauges plotted in the relevant figures together with corresponding F. Figure 23 presents the comparison of F–CMOD and F–CTOD curves for some of the samples (PM_3_2; PD_3_2; FF_2_3). Additionally, on the left side of Figure 23, the localization of CMOD and CTOD measurements in the software is shown. It can be concluded that comparing F–CMOD and F–CTOD, better correlation is achieved for CTOD and curves overlapping each other almost perfectly. The CMOD fit is also very good, however in the phase after the crack occurrence, some small shifts are observed. This can be due to the fact that CMOD in the DIC system was measured in the place not exactly corresponding with the localization of the clip gauge position, and with the increasing value of CMOD, the error is higher and more visible. Nevertheless, the fit accuracy for both CMOD and CTOD presented in Figure 23 and the rest of the specimens was almost perfect. It can be concluded then that the DIC technique can successfully replace and/or support the traditional methods for measuring the displacements during 3PBT. Additionally, in Table 14 the crack propagation and ε_x_ strain maps are presented for selected CMOD, corresponding with $f_{\mathrm{ct},L}^{f}$ and f_R,1_–f_R,4_. It is easily visible that the crack propagation developed within increasing F and CMOD. The conclusion mentioned before that the beams were destroyed by a quasi-vertical crack beginning in the sample notch is also confirmed by the DIC technique. When it comes to strain maps, only for small CMOD up to 0.5 mm the horizontal strains could have been noticed in the images. When the crack width was increasing, two parts of the sample were detaching from each other, making the camera not able to recognize the speckle pattern.

## 4. Conclusions

The article presents the possibility of using the EN 14651 standard to determine the flexural tensile strength of concrete with the addition of 2 and 3 kg/m^3^ of synthetic fibers of different geometry and form. The following conclusions were drawn from the conducted research:

- As a result of adding synthetic fibers to concrete, the workability of the mixes deteriorated significantly. The consistency class decreased by three for FF_2, two for PM_3, PD_3 and PD_2, and one for PM_2, compared to plain concrete. Nevertheless, the type and fiber content did not significantly affect the compressive strength, which ranged from 58.06 to 61.31 MPa, depending on the mixture, while for concrete without fibers it was equal to 58.05 MPa.
- The fibers contributed to an increase in the flexural tensile strength of approximately 5.5% for PM_3, PM_2, and PD_3; 11.5% for PD_2 and 13.5% for FF_2. The mixtures with slightly shorter fibers (l_f_ = 48 mm) performed better, and in particular the one where a smaller amount of fibers (2 kg/m^3^) has been used. However, the best results were obtained with a hybrid blend with V_f_ = 2 kg/m^3^. This may be due to the fact that the combination of two types of fibers more effectively bridged the microcracks, and the lower fiber content allowed fibers for their more even distribution in the concrete.
- The damage of beams without fibers was brittle and sudden, unlike for the fiber-reinforced concrete samples, which were still able to transfer a significant load with increasing crack mouth opening displacement, and possessed some ductility. The residual flexural tensile strengths of concrete with synthetic fibers usually reached higher values, when the fiber content was higher. Additionally, the mixture with the incorporation of the larger amount of fibers which were longer (l_f_ = 54 mm) performed best. It was because longer fibers more efficiently bridged the macrocracks. It is also worth noting that among the mixtures with 2 kg/m^3^, the highest values for almost all residual strengths were achieved for the hybrid blend, which may indicate the positive effect of combining two different types of fibers.
- As a result of the addition of fibers to concrete, the fracture energy, calculated up to the crack mouth opening displacement of 3.5 mm, increased from about 16 to 22 times depending on the type of the mixture.
- Significant dispersion of the force-crack mouth opening displacement curves was the result of a small fracture area (high statistical variability of the number of fibers crossing this surface) resulting from the used test method-according to EN 14651. Additionally, in the case of some mixtures, it was noticeable that some of the twisted fibers did not disintegrate, which resulted in the uneven distribution of fibers. The reason for large deviations in the results of individual samples may be connected with a different number of fibers in the cross-section of the crack.
- The formula proposed by the authors for calculating the flexural tensile strength for concrete with polymer fibers with a nominal fiber content of ≤ 1.0% and slenderness up to 200 correlates very well with the results of the presented studies, as well as with those contained in various literature positions. Regarding the formula proposed in the EN 14651 standard to determine the relationship between the deflection and the crack mouth opening displacement, it was concluded that it differs from the relationship recorded during the presented tests of beams with synthetic fibers. As a result, a new formula was proposed by the authors, together with equations describing crack mouth opening displacement–crack tip opening displacement and crack tip opening displacement–deflection dependencies.
- According to Model Code 2010, the use of 2 and 3 kg/m^3^ of tested fibers in the concrete mix would not partially or fully replace traditional reinforcement in a form of steel bars. Furthermore, it was not possible to obtain σ-w curves from uniaxial tensile tests, and received results for maximum tensile strength did not show the clear influence of fibers’ incorporation on concrete.
- The DIC technique can be successfully used to determine the crack mouth and crack tip opening displacements with very high accuracy.

## Figures and Tables

**Table 1:** Research about the influence of synthetic FRC.

Ref.	Workability	Compressive Strength	Flexural Tensile Strength acc. to EN 14651 [28]	Fracture Energy	Uniaxial Tensile Strength	Model Code Classification [26]	DIC System
[13]	x	x	x ^1^				
[22]		x	x ^1^		x		
[23]	x	x	x ^1^	x			
[38]		x					x
[40]		x	x	x			x
[41]		x	x	x			x
[46]	x	x	x			x	
[47]	x	x	x			x	
[48] ^2^	x	x	x			x	
[50]	x	x	x ^3^				
[51]		x	x ^1^				
[52]		x	x ^1^				
[53]	x	x	x ^1^	x			
[54]					x		
[55]		x			x		
[56] ^2^					x		
This study	x	x	x	x	x	x	x

^1^ Acc. to different standard. ^2^ Research on steel FRC. ^3^ No information about the standard.

**Table 2:** Characterization of fiber properties.

	PM	PD	FF
l_f_	54 mm	48 mm	52 mm/52 mm
d_f_	0.45 mm	0.60 mm	0.45 mm/-
l_f_/d_f_	120	80	115/-
f_t_	550–650 MPa	550–600 MPa	620–758 MPa
Material	copolymer	copolymer	95% copolymer/5% polypropylene
Form	twisted, monofilament	monofilament	twisted, monofilament/fibrillated
	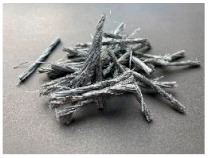	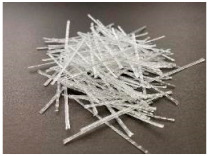	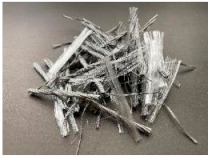

**Table 3:** Concrete mixture composition [kg/m^3^].

Composition	PC	PM_3	PM_2	PD_3	PD_2	FF_2
CEM 42.5R	400					
Pebble aggregate 2–8 mm	1052					
Sand 0–2 mm	673					
Water	200					
Superplasticizer	1.43					
w/c	0.50					
Fibers	0.0	3.0	2.0	3.0	2.0	2.0
V_f_	0.00%	0.33%	0.22%	0.33%	0.22%	0.22%

**Table 4:** Mixing procedure.

Procedure	Mixing Time
50% of pebble aggregate + 50% of sand	1 min
50% of CEM I 42.5R	1 min
100% of water + 100% of superplasticizer	2 min
50% of pebble aggregate + 50% of sand + 50% of CEM 42.5R	1 min
100% of fibers	2 min ^1^ + * + 1 min + * + 1 min

^1^ Only for mixes with fibers. * Technical break to remove material on the walls and blades of the mixer.

**Table 5:** Slump classification according to EN 12350-2 [58].

-	PC	PM_3	PM_2	PD_3	PD_2	FF_2
h [mm]	230	130	160	140	140	65
Consistency class	S5	S3	S4	S3	S3	S2

**Table 6:** Research program–number of studied samples for performed tests.

Type of the Test	PC	PM_3	PM_2	PD_3	PD_2	FF_2
Compressive strength test	6	6	6	6	6	6
Flexural tensile test	3	3	3	3	3	3
Uniaxial tensile test	1	1	1	1	1	1

**Table 7:** Compressive strength for all samples for individual concrete mixes.

No.	Property	PC			PM_3			PM_2		
1	f_c_ [MPa]	57.49	f_cm_ [MPa]	58.05	56.61	f_cm_ [MPa]	58.06	61.50	f_cm_ [MPa]	60.64
2		58.40	s_fc_ [MPa]	1.33	58.65	s_fc_ [MPa]	1.10	64.57	s_fc_ [MPa]	2.20
3		59.89	V_fc_ [%]	2.29	57.23	V_fc_ [%]	1.89	58.91	V_fc_ [%]	3.64
4		58.21	f_c;0.05_ [MPa]	57.26	59.27	f_c;0.05_ [MPa]	57.34	60.03	f_c;0.05_ [MPa]	59.14
5		63.92			58.52			58.48		
6		56.26			32.85			60.34		
No.	Property	PD_3			PD_2			FF_2		
1	f_c_ [MPa]	56.71	f_cm_ [MPa]	59.44	59.79	f_cm_ [MPa]	61.31	60.73	f_cm_ [MPa]	60.87
2		59.59	s_fc_ [MPa]	2.77	64.10	s_fc_ [MPa]	1.81	60.71	s_fc_ [MPa]	0.72
3		60.45	V_fc_ [%]	4.66	62.02	V_fc_ [%]	2.95%	61.93	V_fc_ [%]	1.19
4		61.80	f_c;0.05_ [MPa]	57.55	60.84	f_c;0.05_ [MPa]	60.23	61.43	f_c;0.05_ [MPa]	60.38
5		55.57			55.54			60.59		
6		62.50			59.81			59.84		

**Table 8:** Force values corresponding to the limit of proportionality (F_L_) and CMOD = 0.5 (F_1_); 1.5 (F_2_); 2.5 (F_3_) and 3.5 mm (F_4_) for tested concrete mixtures.

Ref.	PC	PM_3	PM_2	PD_3	PD_2	FF_2
F_L_ [kN]	10.17	10.74	10.70	10.75	11.34	11.55
F_1_ [kN]	-	4.74	3.34	3.62	6.15	3.78
F_2_ [kN]	-	3.33	2.30	2.91	2.15	2.61
F_3_ [kN]	-	3.29	2.09	2.90	2.27	2.53
F_4_ [kN]	-	3.53	2.12	2.88	2.25	2.43

**Table 9:** The flexural tensile strength of FRC calculated using empirical formulas and resulting from the tests carried out.

Mixture	f_fl_ acc. to Equation (5) [MPa]	f_fl_ acc. to Equation (6) [MPa]	f_fl_ acc. to Equation (7) [MPa]	$f_{\mathrm{ct},L}^{f}=f_{\mathrm{fl}}\;\mathrm{from}\;\mathrm{Studies}\;[\mathrm{MPa}]$
PM_3	7.43 (2.16)	3.92 (1.14)	4.50 (1.31)	3.44
PM_2	7.59 (2.22)	2.86 (0.83)	4.05 (1.18)	3.42
PD_3	7.45 (2.17)	2.86 (0.83)	4.05 (1.18)	3.44
PD_2	7.68 (2.12)	2.15 (0.59)	3.75 (1.03)	3.63
FF_2	7.70 (2.08)	2.78 (0.75)	4.02 (1.09)	3.69

Note: the ratio of the calculated f_fl_ from the given formula to that resulting from the performed studies is presented in the brackets.

**Table 10:** Comparison of the results of the presented research and the results from selected literature positions with the results obtained for the empirical formula proposed by the authors for the flexural tensile strength f_fl_.

Ref.	V_f_ [%]	l_f_ [mm]	d_f_ [mm]	l_f_/d_f_	f_fl_ [MPa]	f_fl_ acc. to Equation (8) [MPa]	f_fl_ acc. to Equation (8)/f_fl_
Presented research	0.00	-	-	-	3.26	3.26	1.000
	0.33	54	0.45	120	3.44	3.52	1.024
	0.22	54	0.45	120	3.42	3.43	1.002
	0.33	48	0.60	80	3.44	3.43	0.997
	0.22	48	0.60	80	3.63	3.37	0.929
	0.22	52	0.45	115	3.69	3.43	0.927
[69]	0.00	-	-	-	4.36	4.36	1.000
	0.30	50	0.660	76	4.64	4.51	0.971
	0.33	50	0.660	76	4.54	4.52	0.997
[10]	0.00	-	-	-	7.70	7.70	1.000
	0.50	12	0.025	480	8.00	9.34	1.168
	1.00	12	0.025	480	7.00	10.98	1.569
	1.50	12	0.025	480	6.50	12.62	1.942
	2.00	12	0.025	480	5.60	14.27	2.548
[13]	0.00	-	-	-	3.95	3.95	1.000
	0.22	50	1.000	50	3.77	4.02	1.066
	0.33	50	1.000	50	3.94	4.05	1.029
	0.43	50	1.000	50	3.88	4.08	1.052
[77]	0.00	-	-	-	4.20	4.20	1.000
	0.51	40	0.706 *	57	4.40	4.38	0.996
	0.59	40	0.706 *	57	4.45	4.41	0.991
[78]	0.00	-	-	-	5.54	5.54	1.000
	0.50	15	0.100	150	5.74	6.04	1.052
[79]	0.00	-	-	-	4.78	4.78	1.000
	0.30	15	0.100	150	4.69	5.08	1.084
[80]	0.00	-	-	-	5.21	5.21	1.000
	0.50	20	0.100	200	5.61	5.88	1.049
[68]	0.00	-	-	-	4.58	4.58	1.000
	0.33	60	1.000	60	4.87	4.71	0.967
	0.67	60	1.000	60	5.23	4.83	0.925
[70]	0.00	-	-	-	2.20	2.20	1.000
	0.20	55	0.850	65	2.27	2.29	1.009
	0.40	55	0.850	65	2.30	2.37	1.033
	0.60	55	0.850	65	2.25	2.46	1.092
[74]	0.00	-	-	-	3.09	3.09	1.000
	0.70	60	0.580	103	3.44	3.58	1.039
	1.00	60	0.580	103	3.40	3.78	1.113
	0.00	-	-	-	3.79	3.79	1.000
	0.70	60	0.580	103	4.11	4.27	1.039
	1.00	60	0.580	103	4.32	4.48	1.036
[81]	0.00	-	-	-	4.73	4.73	1.000
	0.32	40	0.354 *	113	4.69	4.97	1.059
	0.48	40	0.354 *	113	4.82	5.09	1.055
[65]	0.00	-	-	-	4.33	4.33	1.000
	0.80	48	0.917	52	5.09	4.59	0.902
	0.60	48	0.917	52	4.49	4.52	1.008
	0.40	48	0.917	52	4.36	4.46	1.022
[82]	0.00	-	-	-	2.62	2.62	1.000
	0.15	12	0.020	600	2.64	3.25	1.230
	0.30	12	0.020	600	2.84	3.87	1.363
	0.50	12	0.020	600	3.32	4.71	1.418
[47]	0.00	-	-	-	5.00	5.00	1.000
	0.40	48	0.900	53	5.00	5.13	1.026
	0.60	48	0.900	53	5.60	5.19	0.928
	0.80	48	0.900	53	5.00	5.26	1.052
	1.00	48	0.900	53	5.10	5.32	1.044
	1.50	48	0.900	53	5.70	5.49	0.962
[83]	0.00	-	-	-	5.60	5.60	1.000
	0.22	19	0.095	200	5.91	5.90	0.998

* Equivalent fiber diameter was calculated because the cross-section of the fiber was rectangular.

**Table 11:** Principles of FRC classification according to Model Code 2010 [26].

Parameter 1:	Parameter 2:		Total or Partial Replacement of Traditional Reinforcement Is Possible When:
Two consecutive numbers in series 1.0; 1.5; 2.0; 2.5; 3.0; 4.0; 5.0; 6.0; 7.0; 8.0; … [MPa] define the strength range f_R,1k_.	*a*	for 0.5 ≤ f_R,3k_/f_R,1k_ < 0.7	f_R,1k_/$f_{\mathrm{ct},\mathrm{Lk}}^{f}$ > 0.4 and
	*b*	for 0.7 ≤ f_R,3k_/f_R,1k_ < 0.9	f_R,3k_/f_R,1k_ > 0.5
	*c*	for 0.9 ≤ f_R,3k_/f_R,1k_ < 1.1	
	*d*	for 1.1 ≤ f_R,3k_/f_R,1k_ < 1.3	
	*e*	for 1.3 ≤ f_R,3k_/f_R,1k_	

**Table 12:** FRC classification according to Model Code 2010 [26].

PM_3									
$f_{\mathrm{ct},\mathrm{Lm}}^{f}$ [MPa]	3.44	$S_{\mathrm{ct},L}^{f}$ [MPa]	0.37	k	1.89	$f_{\mathrm{ct},\mathrm{Lk}}^{f}$ [MPa]	2.74	f_R,3k_/f_R,1k_	0.95
f_R,1m_ [MPa]	1.52	s_R,1_ [MPa]	0.61		1.89	f_R,1k_ [MPa]	0.36	f_R,1k_/$f_{\mathrm{ct},\mathrm{Lk}}^{f}$	0.13
f_R,3m_ [MPa]	1.05	s_R,3_ [MPa]	0.38		1.89	f_R,3k_ [MPa]	0.34		
**PM_2**									
$f_{\mathrm{ct},\mathrm{Lm}}^{f}$ [MPa]	3.42	$S_{\mathrm{ct},L}^{f}$ [MPa]	0.42	k	1.89	$f_{\mathrm{ct},\mathrm{Lk}}^{f}$ [MPa]	2.64	f_R,3k_/f_R,1k_	0.43
f_R,1m_ [MPa]	1.07	s_R,1_ [MPa]	0.20		1.89	f_R,1k_ [MPa]	0.68	f_R,1k_/$f_{\mathrm{ct},\mathrm{Lk}}^{f}$	0.26
f_R,3m_ [MPa]	0.67	s_R,3_ [MPa]	0.20		1.89	f_R,3k_ [MPa]	0.30		
**PD_3**									
$f_{\mathrm{ct},\mathrm{Lm}}^{f}$ [MPa]	3.44	$S_{\mathrm{ct},L}^{f}$ [MPa]	0.31	k	1.89	$f_{\mathrm{ct},\mathrm{Lk}}^{f}$ [MPa]	2.86	f_R,3k_/f_R,1k_	0.13
f_R,1m_ [MPa]	1.16	s_R,1_ [MPa]	0.25		1.89	f_R,1k_ [MPa]	0.69	f_R,1k_/$f_{\mathrm{ct},\mathrm{Lk}}^{f}$	0.24
f_R,3m_ [MPa]	0.93	s_R,3_ [MPa]	0.44		1.89	f_R,3k_ [MPa]	0.09		
**PD_2**									
$f_{\mathrm{ct},\mathrm{Lm}}^{f}$ [MPa]	3.63	$S_{\mathrm{ct},L}^{f}$ [MPa]	0.10	k	1.89	$f_{\mathrm{ct},\mathrm{Lk}}^{f}$ [MPa]	3.43	f_R,3k_/f_R,1k_	0.66
f_R,1m_ [MPa]	1.97	s_R,1_ [MPa]	0.71		1.89	f_R,1k_ [MPa]	0.63	f_R,1k_/$f_{\mathrm{ct},\mathrm{Lk}}^{f}$	0.18
f_R,3m_ [MPa]	0.73	s_R,3_ [MPa]	0.16		1.89	f_R,3k_ [MPa]	0.42		
**FF_2**									
$f_{\mathrm{ct},\mathrm{Lm}}^{f}$ [MPa]	3.69	$S_{\mathrm{ct},L}^{f}$ [MPa]	0.11	k	1.89	$f_{\mathrm{ct},\mathrm{Lk}}^{f}$ [MPa]	3.48	f_R,3k_/f_R,1k_	0.39
f_R,1m_ [MPa]	1.21	s_R,1_ [MPa]	0.04		1.89	f_R,1k_ [MPa]	1.14	f_R,1k_/$f_{\mathrm{ct},\mathrm{Lk}}^{f}$	0.33
f_R,3m_ [MPa]	0.81	s_R,3_ [MPa]	0.19		1.89	f_R,3k_ [MPa]	0.45		

**Table 13:** Standard deviation, coefficient of variation of fracture energy and toughness index for individual concrete mixes.

Parameter	PC	PM_3	PM_2	PD_3	PD_2	FF_2
s_GF-CMOD=3.5_ [Nm]	0.18	4.27	2.89	4.54	1.38	1.73
V_GF-CMOD=3.5_ [%]	27.56%	30.58%	28.10%	36.50%	11.75%	14.56%
T_i_ [-]	1.0	21.97	16.19	19.59	18.56	18.71

**Table 14:** Comparison of ε_x_ strain maps and crack propagation in FRC beams at considered CMOD.

CMOD [mm]	0.05→$f_{\mathrm{ct},L}^{f}$	0.5→f_R,1_	1.5→f_R,2_	2.5→f_R,3_	3.5→f_R,4_	Contour Legend
PM_3_1					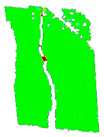	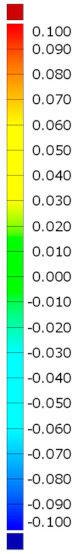
PM_3_2			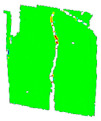	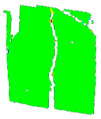	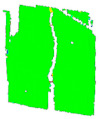	
PM_3_3		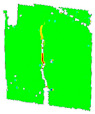	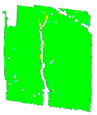	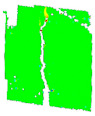	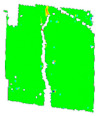	
PM_2_1	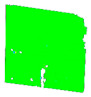	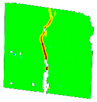	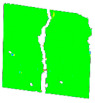	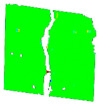	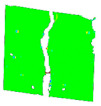	
PM_2_2			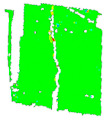	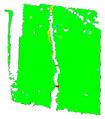	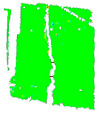	
PM_2_3	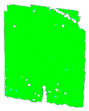	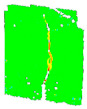	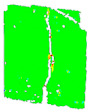	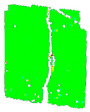	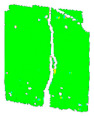	
PD_3_1	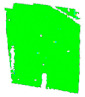	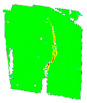	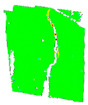	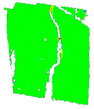	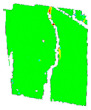	
PD_3_2	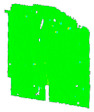	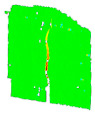	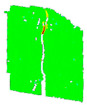	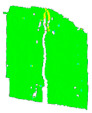	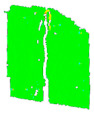	
PD_3_3	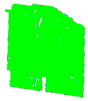	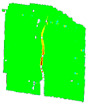	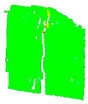	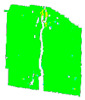	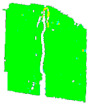	
PD_2_1	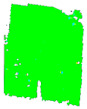	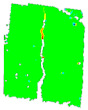	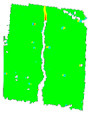	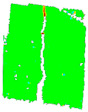	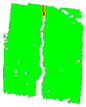	
PD_2_2	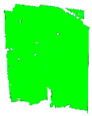	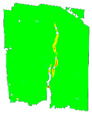	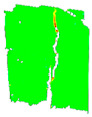	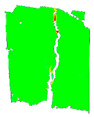	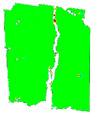	
PD_2_3	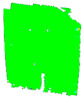		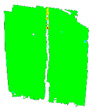	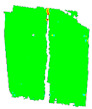	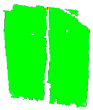	
FF_2_1	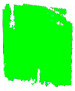	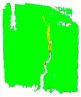	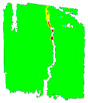	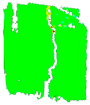	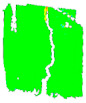	
FF_2_2	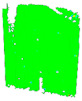	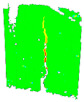	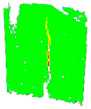	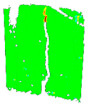	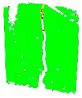	
FF_2_3	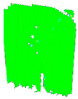	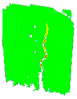	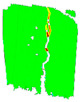	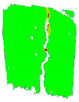	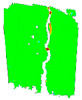	

Note: Empty cells in the table are due to the fact that in some stages the software was not able to process the data.

## Data Availability

Not applicable.
